# Dysbiosis in Metabolic Genes of the Gut Microbiomes of Patients with an Ileo-anal Pouch Resembles That Observed in Crohn's Disease

**DOI:** 10.1128/mSystems.00984-20

**Published:** 2021-03-02

**Authors:** Vadim Dubinsky, Leah Reshef, Keren Rabinowitz, Karin Yadgar, Lihi Godny, Keren Zonensain, Nir Wasserberg, Iris Dotan, Uri Gophna

**Affiliations:** a Shmunis School of Biomedicine and Cancer Research, George S. Wise Faculty of Life Sciences, Tel-Aviv University, Tel Aviv, Israel; b Division of Gastroenterology, Rabin Medical Center, Petah-Tikva, Israel; c Felsenstein Medical Research Center, Rabin Medical Center, Petah Tikva, Israel; d Sackler Faculty of Medicine, Tel-Aviv University, Tel Aviv, Israel; e Colorectal Unit, Division of Surgery, Rabin Medical Center, Petah-Tikva, Israel; Johns Hopkins Bloomberg School of Public Health

**Keywords:** pouchitis, UC, CD, mucin, butyrate, bile acids, oxidative stress, classifier, Crohn's disease, microbiome, ulcerative colitis

## Abstract

Crohn's disease (CD), ulcerative colitis (UC), and pouchitis are multifactorial and chronic inflammatory bowel diseases (IBD). Pouchitis develops in former UC patients after proctocolectomy and ileal-pouch-anal anastomosis and is characterized by inflammation of the previously normal small intestine comprising the pouch. The extent to which microbial functional alteration (dysbiosis) in pouchitis resembles that of CD or UC has not been investigated, and the pathogenesis of pouchitis remains unknown. We collected 208 fecal metagenomes from 69 patients with a pouch (normal pouch and pouchitis) and compared them to publicly available metagenomes of patients with CD (*n* = 88), patients with UC (*n* = 76), and healthy controls (*n* = 56). Patients with pouchitis presented the highest alterations in species, metabolic pathways, and enzymes, which was correlated with intestinal inflammation. Ruminococcus gnavus strains encoding mucin-degrading glycoside hydrolases were highly enriched in pouchitis. Butyrate and secondary bile acid biosynthesis pathways were decreased in IBD phenotypes and were especially low in pouchitis. Pathways such as amino acid biosynthesis and degradation of aromatic compounds and sugars, encoded by members of the *Enterobacteriaceae*, were enriched in pouch and CD but not in UC. We developed microbial feature-based classifiers that can distinguish between patients with a normal pouch and pouchitis and identified species and genes that were predictive of pouchitis. We propose that the noninflamed pouch is already dysbiotic and microbially is similar to CD. Our study reveals microbial functions that outline the pathogenesis of pouchitis and suggests bacterial groups and functions that could be targeted for intervention to attenuate small intestinal inflammation present in pouchitis and CD.

**IMPORTANCE** Crohn's disease (CD), ulcerative colitis (UC), and pouchitis are chronic inflammatory conditions of the bowel. Pouchitis develops in former UC patients after proctocolectomy and ileal-pouch-anal anastomosis and is characterized by inflammation of the previously normal small intestine comprising the pouch. The extent to which microbial dysbiosis in patients with pouchitis resembles that of CD or UC and the pathogenesis of pouchitis remains unclear. We investigated the functions in the gut microbiomes of these patients using metagenomics. We found that the noninflamed pouch is already dysbiotic and microbially is similar to CD. Our study reveals microbial functions with a potential role in pouchitis pathogenesis such as depletion in butyrate and secondary bile acid synthesis and enrichment of amino acid synthesis and degradation of aromatic compounds, encoded by members of the *Enterobacteriaceae*. We developed microbial feature-based classifiers that can distinguish between patients with a normal pouch and pouchitis and identified species and genes that were predictive of pouchitis. We suggest species and functions that could be targeted for intervention to attenuate small intestinal inflammation present in pouchitis and CD.

## INTRODUCTION

Inflammatory bowel diseases (IBD), including Crohn's disease (CD), ulcerative colitis (UC), and pouchitis, are chronic, relapsing, and remitting inflammatory conditions of the intestine. CD mainly affects the small intestine and colon, while UC is localized to the colon. The etiology of IBD is multifactorial, involving genetic predisposition (1), environmental triggers (2), and an aberrant immune-mediated response to specific antigens of the intestinal microbiota (3).

Approximately 25% of patients with complicated UC may undergo total large bowel resection. Reconstruction of intestinal continuity is achieved by the creation of a reservoir (“pouch”) from the healthy small intestine which is connected to the anus (ileal pouch-anal anastomosis, also called pouch surgery) (4). Up to 70% of these former UC patients may develop inflammation of the previously normal small intestine comprising the pouch (pouchitis) (5). Pouchitis is the most common complication after pouch surgery, but its pathogenesis is still largely unknown (6).

It was shown previously that the gut microbiome in patients with IBD is characterized by lower diversity, lower stability, and compositional changes in specific taxa compared to that in healthy individuals (7). Levels of facultative anaerobes from the *Enterobacteriaceae* are increased, while beneficial obligate anaerobes, such as Faecalibacterium prausnitzii and Eubacterium rectale, are decreased (8–12). Shifts in microbial functions in the metagenomes of patients with IBD have also been demonstrated, e.g., lower presence of short-chain fatty acid (SCFA) synthesis pathways, increase in oxidative stress response genes, and alteration in amino acid metabolism genes (9, 13). These taxonomic and functional changes are broadly referred to as dysbiosis.

Since most patients with a pouch will develop inflammation in what has been normal uninflamed small bowel tissue (5), these patients represent a unique condition modeling the development of *de novo* small intestinal inflammation, such as that characterizing ileal CD (14). We demonstrated previously that patients with a pouch and CD might have several mechanisms of pathogenesis in common, including mucosal gene and microRNA expression (14, 15), antiglycan serologic responses (16), and CD-like bacterial dysbiosis (11). However, the extent to which microbial dysbiosis in pouchitis resembles that of CD has not been investigated in depth. Here, we analyzed the shotgun metagenomes of patients with a pouch, patients with IBD, and healthy subjects with the following aims. (i) We wanted to identify microbial signatures (species and functional genes) that might be common to disease phenotypes and thus to place patients with a pouch in the context of “primary” IBD. More specifically, we wanted to test whether pouchitis has microbial features that are most similar to those of CD. (ii) We wanted to discover microbial features which might be predictive of pouchitis (untargeted approach). (iii) We wanted to examine key bacterial functions that may underlie the pathogenesis of pouchitis: excessive mucin degradation, reduced butyrate and secondary bile acids production, and increased bacterial resistance to oxidative stress.

## RESULTS AND DISCUSSION

We enrolled 69 patients with a pouch in this study; the median age was 43.8 years (interquartile range, 33.8 to 62.2 years), and 60% were males (Data Set S1, sheet 1). Two hundred eight fecal samples were collected longitudinally (median of 3 samples per patient) over a period of up to 5.8 years. Based on our classification system, these patients were divided into two groups (discovery cohort): patients with a normal pouch (noninflamed; *n* = 35 patients; 103 samples) and those with pouchitis (inflamed; *n* = 34; 105 samples). We also used 20 additional patients with a pouch from our center (Rabin Medical Center [RMC]) and 15 pouch patients from an American cohort (17) as a validation cohort (Data Set S1, sheet 2).

10.1128/mSystems.00984-20.10 DATA SET S1 (Sheet 1) Clinical metadata for patients with a pouch (discovery cohort). (Sheet 2) Clinical metadata for patients with a pouch (validation cohort). (Sheet 3) Clinical metadata for PRISM-DEEP-NLIBD cohorts. (Sheet 4) Linear mixed-effect model of species composition. (Sheet 5) Butyrate producers’ taxonomic composition based on the four terminal genes for butyrate biosynthesis (*but*, *buk*, *ato*, and *4hbt*). (Sheet 6) Secondary bile acid producers based on the *baiA*-*baiI* operon. (Sheet 7) Linear mixed-effect model of enzymes profile. (Sheet 8) Linear mixed-effect model of metabolic pathways. (Sheet 9) Species feature importance scores. (Sheet 10) Pathway feature importance scores. (Sheet 11) Enzyme feature importance scores. (Sheet 12) All Kruskal-Wallis tests pairwise *P* value results. Download Data Set S1, XLSX file, 0.9 MB. Copyright © 2021 Dubinsky et al. 2021 Dubinsky et al. https://creativecommons.org/licenses/by/4.0/ This content is distributed under the terms of the Creative Commons Attribution 4.0 International license.

To explore the microbiomes of patients with a pouch in the context of primary IBD, we analyzed shotgun metagenomes derived from fecal samples of patients with a pouch, UC, and CD and healthy subjects in terms of species composition and functional gene repertoires. Metagenomes of patients with CD (*n* = 88), those with UC (*n* = 76), and healthy subjects (*n* = 56) were obtained from previously published data (18), which are part of the PRISM, LifeLines DEEP, and NLIBD cohorts (cross-sectional) (Data Set S1, sheet 3). To minimize potential biases, raw metagenomic reads from all cohorts were processed in parallel through the same bioinformatic pipeline, in line with recent recommendations for multicohort studies (19).

### Microbial dysbiosis across disease phenotypes and its association with inflammation.

**FIG 1:**
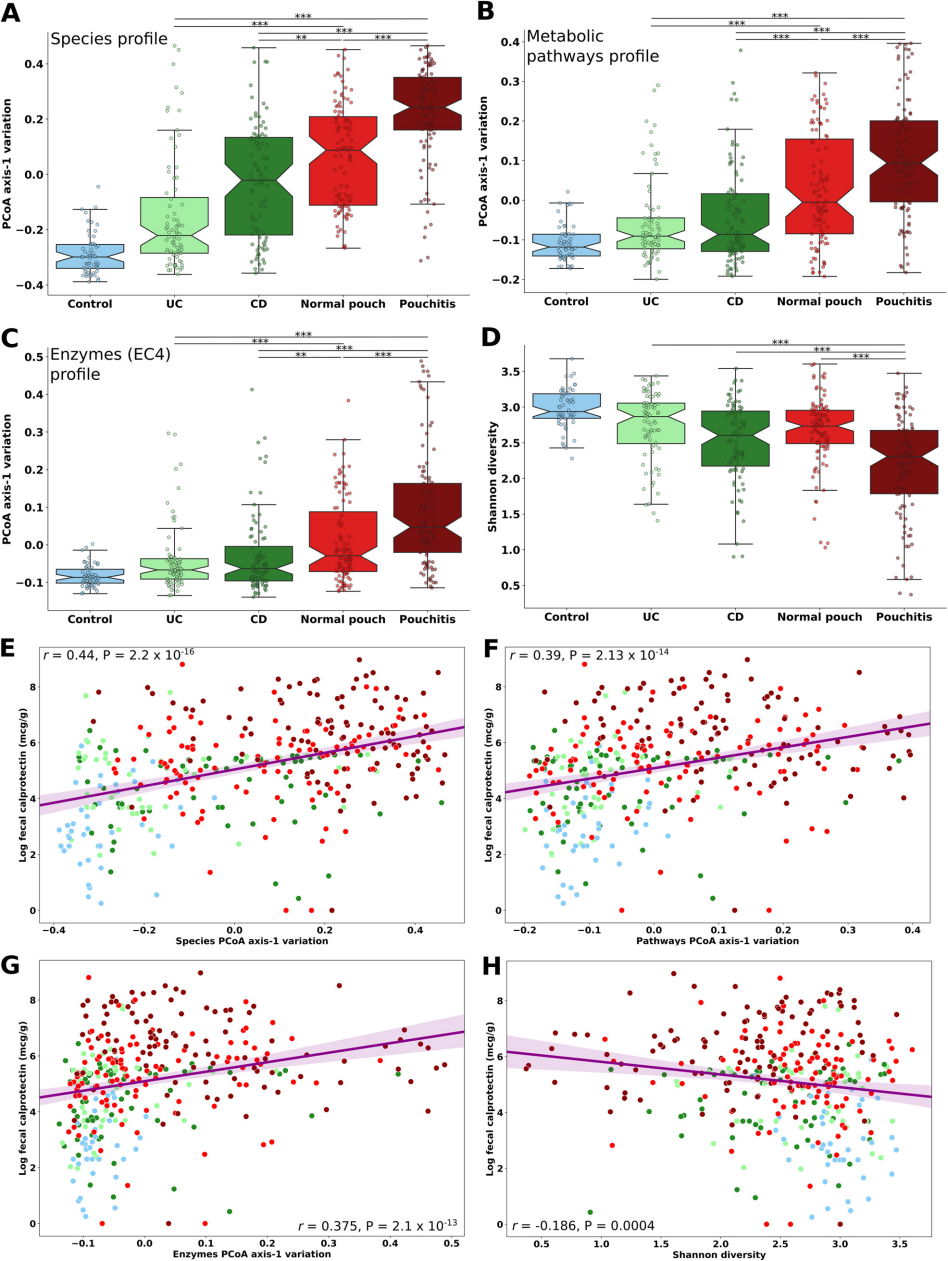
Microbial community stratification among IBD disease phenotypes and healthy subjects. (A to C) Microbiome variation according to the first axis of principal-coordinate analysis (PCoA) of (A) species, (B) metabolic pathways, and (C) enzyme profiles, based on Bray-Curtis dissimilarity of the fecal metagenomes. (D) Shannon diversity in the gut microbiome based on species composition. (E to H) Spearman correlation calculated between fecal calprotectin level (log) and the first axis of microbiome variation for species (E), metabolic pathways (F), enzymes (G), and Shannon diversity (H). Lines are linear regression fitted with a 95% confidence interval. Colors represent the different IBD phenotypes (UC, CD, normal pouch, and pouchitis), as well as healthy subjects (control). *, *P* < 0.05; **, *P* < 0.01; ***, *P* < 0.001; Kruskal-Wallis, applying Dunn’s multiple correction test. For a list of all pairwise *P* values, see Data Set S1, sheet 12. Box plot whiskers mark observations within the 1.5 interquartile range of the upper and lower quartiles.

The microbial community composition of disease phenotypes and that of healthy subjects (beta-diversity, analyzed by principal-coordinate analysis [PCoA]) were well separated according to profiles of species, genes, and pathways (Fig. 1A to C; Fig. S1A to C), with some degree of overlap, in particular between samples from UC and CD patients, as was observed previously (18). Strikingly, we observed a significant stratification (Kruskal-Wallis, *P* < 0.01) of different disease phenotypes based on the first axis of microbiome variation, shifting away from the healthy subjects, in the following order: UC, CD, normal pouch, and pouchitis (Fig. 1A to C). The latter was the most distant from the microbiomes of healthy controls; i.e., it had the highest level of dysbiosis. Interestingly, according to Shannon diversity (Fig. 1D), no significant difference was observed between patients with CD (median Shannon diversity index, 2.6) and UC (median Shannon diversity index, 2.87) to patients with a normal pouch (median Shannon diversity index, 2.73). In contrast, patients with pouchitis had significantly lower diversity (median Shannon diversity index, 2.31; Kruskal-Wallis, *P* < 0.001) than all other phenotypes.

10.1128/mSystems.00984-20.1 FIG S1 Microbiome variation (principal-coordinate analysis [PCoA]) based on Bray-Curtis dissimilarity for (A) species, (B) pathway, and (C) enzyme compositional profiles of the fecal metagenomes. (D) Clinical variables explaining the variation in microbiome species, pathways and enzymes, analyzed with PERMANOVA. Percentages indicate variance explained by each variable (*R*^2^ values) independently of the other variables. *, *P* < 0.05; **, *P* < 0.01; ***, *P* < 0.001. Download FIG S1, TIF file, 1.4 MB. Copyright © 2021 Dubinsky et al. 2021 Dubinsky et al. https://creativecommons.org/licenses/by/4.0/ This content is distributed under the terms of the Creative Commons Attribution 4.0 International license.

The subject’s clinical phenotype explained a substantial part of the observed variation in microbial composition (beta-diversity based on Bray-Curtis distance) in species, metabolic pathway, and enzymes (Fig. S1D) (permutational multivariate analysis of variance [PERMANOVA); *R*^2^ = 12.1%, 13.2%, and 10.4%, respectively; *P* < 0.001), while patient age and gender (the latter was available only for the pouch cohort) explained only 0.39% to 0.71% and 0.42% to 0.52%, respectively. Antibiotic use (1 month or 6 months off antibiotics) accounted for only 0.45% to 0.62% of the variation. Fecal calprotectin, a biomarker for intestinal inflammation, explained a low yet significant degree of microbial variation (*R*^2^ = 2.5% to 4.1%, *P* < 0.001). Consistent with previous studies of patients with UC and CD (13, 20), intersubject variation explained the highest degree of microbiome variation (Fig. S1D) (PERMANOVA; *R*^2^ = 46.6% to 48.7%; *P* < 0.001) among the longitudinal samples of patients with a pouch cohort. The cohort from which the samples were derived had a considerable effect as well, explaining 8.7% to 11.2% of the variation. Finally, in patients with a pouch, pouch phenotype and disease activity contributed 4.2% to 4.8% and 1.4% to 1.9%, respectively, to the microbiome variation (Fig. S1D). These observations indicate that besides subject and cohort differences, disease type and inflammation severity are the strongest contributors to microbial variability and the resulting dysbiosis.

If dysbiosis is associated with intestinal inflammation, one would expect a correlation between the microbiome variation and inflammation biomarkers. Indeed, we observed a moderate and significant correlation between the fecal calprotectin level and the first axis of microbiome variation for species, metabolic pathways, and enzymes (Fig. 1E to G) (Spearman *r *= 0.44, 0.39, and 0.375, respectively; *P* < 0.001). A weaker negative correlation was detected between calprotectin and Shannon diversity (Fig. 1H) (Spearman *r* = −0.186; *P* = 4.01 × 10^−4^). These data suggest that patients with a normal pouch already show signs of inflammation and dysbiosis that may parallel those observed in CD and that these are intensified in patients with pouchitis. As patients with a pouch had the highest level of calprotectin (Fig. S2A), we wanted to assess how much of the observed dysbiosis in those patients was due to intestinal inflammation level or pouchitis. We divided these patients based on similar levels of calprotectin and analyzed the variation in microbial composition and diversity. Pouch phenotype was the only factor having a statistically significant effect on the microbiome regardless of the level of inflammation (Fig. S2B to E).

10.1128/mSystems.00984-20.2 FIG S2 Fecal calprotectin levels in IBD and healthy controls and the effect of calprotectin and pouchitis on the microbiome of pouch patients. (A) Fecal calprotectin (log transformed) in patients with IBD and healthy controls. (B to E) Effect of intestinal inflammation (fecal calprotectin) and pouchitis on the variation in microbial composition based on (B) species, (C) pathways, (D) enzymes, and (E) Shannon diversity in pouch patients. Pouch samples were divided into three groups based on calprotectin levels (low, ≤200; medium, 200 to 500; high, ≥500). *, *P* < 0.05; **, *P* < 0.01; ***, *P* < 0.001; Kruskal-Wallis, applying Dunn’s multiple correction test. For a list of all pairwise *P* values, see Data Set S1, sheet 12. Box plot whiskers mark observations within the 1.5 interquartile range of the upper and lower quartiles. Fecal calprotectin measurements were available for 358 of 428 samples. Download FIG S2, TIF file, 1.5 MB. Copyright © 2021 Dubinsky et al. 2021 Dubinsky et al. https://creativecommons.org/licenses/by/4.0/ This content is distributed under the terms of the Creative Commons Attribution 4.0 International license.

To rule out the possibility that the observed differences between the pouch patients’ and nonpouch patients’ microbiomes are inflated due to center or country effects, we obtained and analyzed 15 publicly available pouch metagenomes (17) (from an American cohort). Reassuringly, based on microbiome species, pathway, and enzyme variation (Bray-Curtis) and Shannon diversity (Fig. S3A to D), the pouch samples from the American cohort clustered within the range of the pouches from our RMC pouch cohort.

10.1128/mSystems.00984-20.3 FIG S3 Comparison of 15 publicly available pouch metagenomes from an American cohort (17) to the rest of the pouch and nonpouch metagenomes analyzed in the current study. Microbiome variation according to the first axes of PCoA of (A) species, (B) metabolic pathways, and (C) enzymes profiles, based on Bray-Curtis dissimilarity of the fecal metagenomes. (D) Shannon diversity based on species composition. (E to H) Main butyrate synthesis pathways: (E) butyryl-CoA:acetate CoA transferase (*but*), (F) butyrate kinase (*buk*), (G) butyryl-CoA:acetoacetate CoA transferase (*ato*), and (H) butyryl-CoA:4-hydroxybutyrate CoA transferase (*4hbt*) (I) Secondary bile acid (DCA and LCA) formation (*baiA*-*baiI* operon). The samples labeled “American Pouch Cohort” are the pouch metagenomes from the American cohort (17). Download FIG S3, TIF file, 2.2 MB. Copyright © 2021 Dubinsky et al. 2021 Dubinsky et al. https://creativecommons.org/licenses/by/4.0/ This content is distributed under the terms of the Creative Commons Attribution 4.0 International license.

### Bacterial species associated with IBD.

**FIG 2:**
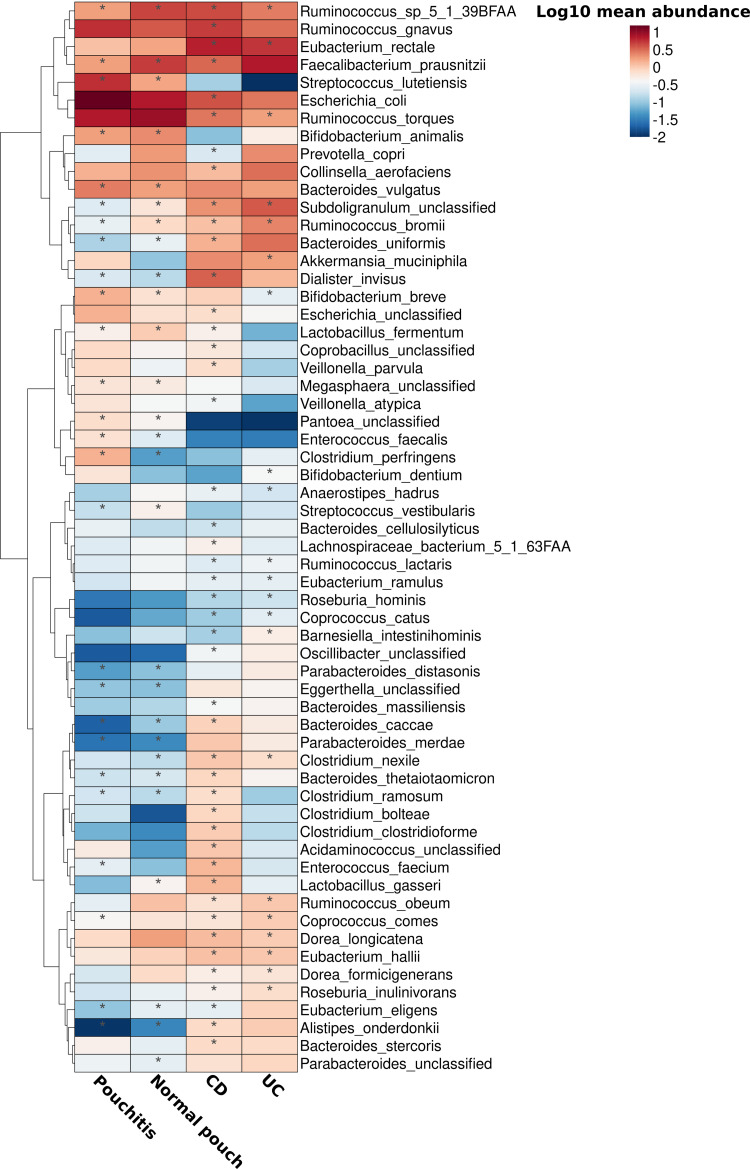
Bacterial species associated with IBD phenotypes. A generalized linear mixed-effects model was built for each bacterial species, and IBD phenotypes were set as a predictor (including age and antibiotic use as fixed effects). Healthy subjects were used as a reference point, and individual subjects and cohort were set as random effects. Only species with a mean relative abundance across all groups of ≥0.15% with at least one significant association with an IBD phenotype are presented. For a full list of species that were significantly associated with IBD phenotypes see Data Set S1, sheet 4. Asterisks indicate patient groups for which the association was significant (FDR < 0.05).

We aimed to characterize the altered species composition in the four IBD groups compared to the composition in healthy subjects, and also to compare patients with a pouch to those with primary IBD, using linear modeling of shotgun metagenomic data. As expected, bacterial species composition showed extensive and significant differences involving many taxa between healthy subjects and patients with UC, with CD, and with a pouch (Fig. 2; Data Set S1, sheet 4). We confirmed past observations of decreased abundance in beneficial taxa such as Faecalibacterium prausnitzii and increased abundance of potential pathobionts such as Escherichia coli in CD and in patients with a pouch (8, 10–12). Patients with a pouch (in particular, those with pouchitis) showed the strongest extremes in terms of the levels of the above-mentioned species, further supporting the existence of a strong dysbiosis in pouchitis.

In addition, we identified significant depletion of an unclassified species of *Subdoligranulum*, a known butyrate-producing genus related to *Faecalibacterium* (21), in all disease phenotypes (Fig. 2), with the lowest mean relative abundance in patients with a normal pouch (0.64%) and those with pouchitis (0.22%). Additionally, Ruminococcus bromii, a keystone species that is highly efficient in the degradation of resistant starch, making its degradation products available for other beneficial bacteria via cross-feeding (22), was highly depleted in all disease phenotypes, especially in patients with a pouch (relative abundances of 0.75% and 0.3% in normal pouch and pouchitis, respectively). Interestingly, we found a trend for the enrichment of Ruminococcus gnavus and Ruminococcus torques in patients with a pouch and for the depletion of Akkermansia muciniphila (Fig. 2). These three species have the potential to degrade human secretory mucin, and an observation of an increase in *R. gnavus* and *R. torques* coupled with a decrease in *A. muciniphila* in biopsy specimens of UC and CD patients was previously reported (23). A higher proportion of mucolytic species in patients with IBD may contribute to increased intestinal inflammation (24). Notably, a distinct clade of *R. gnavus* strains was previously found to be enriched in CD and UC patients and harbored potential virulence-associated genes (25). To critically examine whether the mucin degradation potential of the microbiome is higher in IBD, we moved on to quantify the total mucin-degrading bacterial enzymes in the gut microbiomes of these patients.

### Mucin degradation and *R. gnavus* glycoside hydrolases.

**FIG 3:**
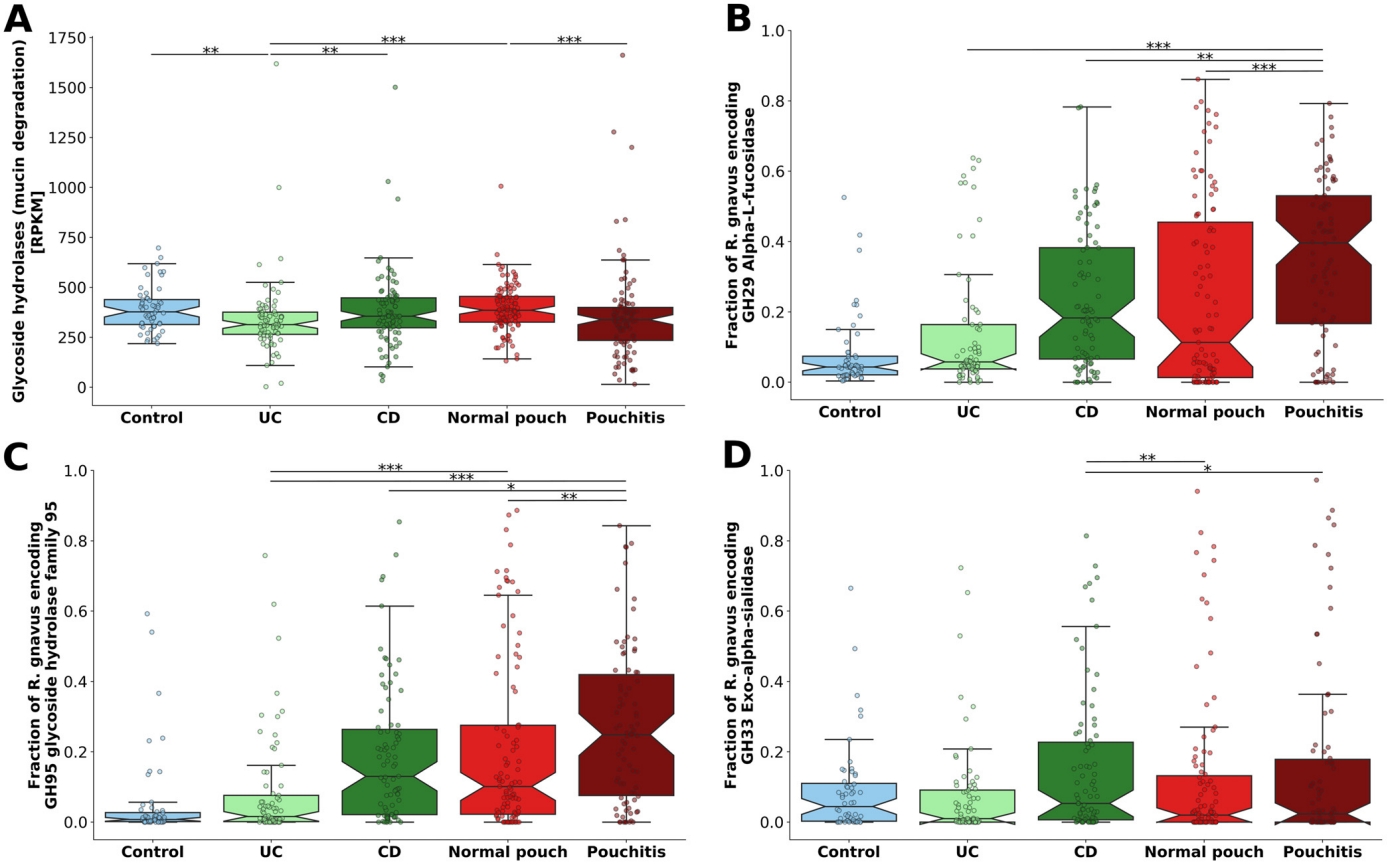
Mucin degradation potential of the microbiome of patients with IBD and healthy controls. (A) Bacterial community mucin degradation potential based on analysis of glycoside hydrolase genes (GH) that are involved in the breakdown and utilization of mucin-derived glycans. (B to D) The relative fraction of Ruminococcus gnavus strains encoding strain-specific glycoside hydrolases (normalized according to three single-copy housekeeping genes) involved in mucin degradation: (B) GH29 α-l-fucosidase, (C) GH95 glycoside hydrolase family 95, and (D) GH33 exo-alpha-sialidase. *, *P* < 0.05; **, *P* < 0.01; ***, *P* < 0.001; Kruskal-Wallis, applying Dunn’s multiple correction test. For a list of all pairwise *P* values, see Data Set S1, sheet 12. Box plot whiskers mark observations within the 1.5 interquartile range of the upper and lower quartiles.

We specifically analyzed glycoside hydrolase (GH) genes that are involved in the breakdown and utilization of mucin-derived glycans and are encoded in the genomes of mucolytic bacteria (Fig. S4). According to these criteria, bacterial community mucin degradation potential in patients with a normal pouch was similar to those of patients with CD and of healthy subjects (median numbers of reads per kilobase of transcript per million mapped reads [RPKM], 386, 338, and 377, respectively; Fig. 3A). Patients with pouchitis had 12.3% lower community mucin degradation potential than patients with a normal pouch (Kruskal-Wallis, *P* = 3.4 × 10^−4^), while patients with UC had lower mucin degradation potential than the other phenotypes (Fig. 3A) (Kruskal-Wallis, *P* < 0.01). These results suggest that the mucin degradation potential of the microbiome does not increase in IBD compared to healthy subjects and even decreases in UC patients.

10.1128/mSystems.00984-20.4 FIG S4 Bacterial community contribution of glycoside hydrolase enzymes that are involved in the breakdown and utilization of mucin-derived glycans by bacterial genera. Each stacked bar on the *x* axis represents a sample from the corresponding phenotype (labels above the bars), and the *y* axis shows the log_10_-scaled total community gene abundance (sum-normalized to copies per million [CPM]). Seven such enzymes were identified and quantified: (A) exo-alpha-sialidase, (B) α-l-fucosidase, (C) β-*N*-acetylhexosaminidase, (D) α-*N*-acetylgalactosaminidase, (E) β-galactosidase, (F) α-*N*-acetylglucosaminidase, and (G) protein *O*-GlcNAcase. The contributions of the top genera are proportionally scaled within the total. See the legend at the bottom of the figure for the color codes. “Other” encompasses contributions from additional lower-abundance genera. The labels over the bars represent the clinical phenotypes: HL, non-IBD; UC, ulcerative colitis; CD, Crohn's disease; NP, normal pouch; and P, pouchitis. Download FIG S4, JPG file, 2.8 MB. Copyright © 2021 Dubinsky et al. 2021 Dubinsky et al. https://creativecommons.org/licenses/by/4.0/ This content is distributed under the terms of the Creative Commons Attribution 4.0 International license.

To better understand the specific role of *R. gnavus* in mucin degradation, we focused on its enzymes that are considered specific for host mucin utilization: GH29 (α-l-fucosidase), GH95 (glycoside hydrolase family 95), and GH33 (exo-alpha-sialidase). The former two can cleave fucose from host mucin glycans, while the latter can cleave terminal sialic acid from sialylated mucins. It was shown that the ability of *R. gnavus* to utilize host mucins is strain dependent and is attributed to a specific GH33 sialidase and to GH29 and GH95 fucosidases (26). Thus, we obtained sequences of GH29, GH95, and GH33 from the representative genome of Ruminococcus gnavus ATCC 29149 (JCM6515) and quantified the abundance of these genes and the fraction of *R. gnavus* strains that possess them in the metagenomes by read mapping (Fig. 3B to D). The fraction of *R. gnavus* strains encoding GH29 and GH95 fucosidases varied extensively across disease phenotypes (Fig. 3B and C) but was significantly higher in patients with pouchitis, with 39.6% and 25% (median) of the strains encoding these fucosidases, respectively (Kruskal-Wallis, *P* < 0.01). No significant difference was observed between patients with a normal pouch (11.3% and 10.1% [median]) and CD (18.3% and 13% [median]) for GH29 and GH95, respectively. Less than 6% of *R. gnavus* strains in UC patients and in healthy subjects encoded these fucosidases. Interestingly, GH33 sialidase was encoded by a significantly smaller proportion of *R. gnavus* strains (Fig. 3D). Patients with a pouch had a similar fraction of *R. gnavu*s-encoded GH33 sialidase (normal pouch, 2%, and pouchitis, 2.4% [medians]; Kruskal-Wallis, *P* = 0.336), which was comparable to values for UC patients and healthy subjects (Kruskal-Wallis, *P* > 0.05). Patients with CD had the highest abundance of *R. gnavus* strains encoding GH33 sialidase (5.3% [median]; Kruskal-Wallis, *P* < 0.05).

A study by Ruseler-van Embden et al. (27) established that the pH level in patients with a normal pouch (5.4) was significantly lower than in patients with pouchitis (6.5). Remarkably, this low pH in the normal pouch substantially inhibited mucin (including fucose residues) degradation. This might explain why we detected a markedly lower proportion of *R. gnavus* strains encoding fucosidases in patients with a normal pouch. Altogether, our findings demonstrate that as a consequence of strong dysbiosis in pouchitis, the overall bacterial community mucin degradation potential is lower, but there is a substantially higher number of mucin-degrading *R. gnavus* strains that might be proinflammatory. Mucin degradation in the normal pouch is similar to that of CD in terms of both microbial community- and *R. gnavus*-associated potential.

### Butyrate synthesis potential of the microbiome is decreased in pouchitis.

Following our finding that patients with a pouch had greatly reduced abundances of beneficial species, some of which are known SCFA producers, we aimed to directly quantify the genes encoding SCFA synthesis enzymes. SCFAs are the major metabolic products of anaerobic fermentation of nondigestible polysaccharides by the human colonic microbiota (28, 29). Here, we focused on butyrate, which is the preferred energy source for colonocytes, improves the gut barrier function, has immunomodulatory and anti-inflammatory properties, and reduces oxidative stress in the colon (28). Butyrate-producing species are found among many non-butyrate-producing species in two dominant families of *Firmicutes*, *Ruminococcaceae* and *Lachnospiraceae* (29). Four main pathways are known for butyrate production: the acetyl coenzyme A (acetyl-CoA), glutarate, 4-aminobutyrate, and lysine pathways. Acetyl-CoA is the dominant pathway for butyrate synthesis and is present in the majority of butyrate producers, and *but* is the most prevalent terminal gene (30).

**FIG 4:**
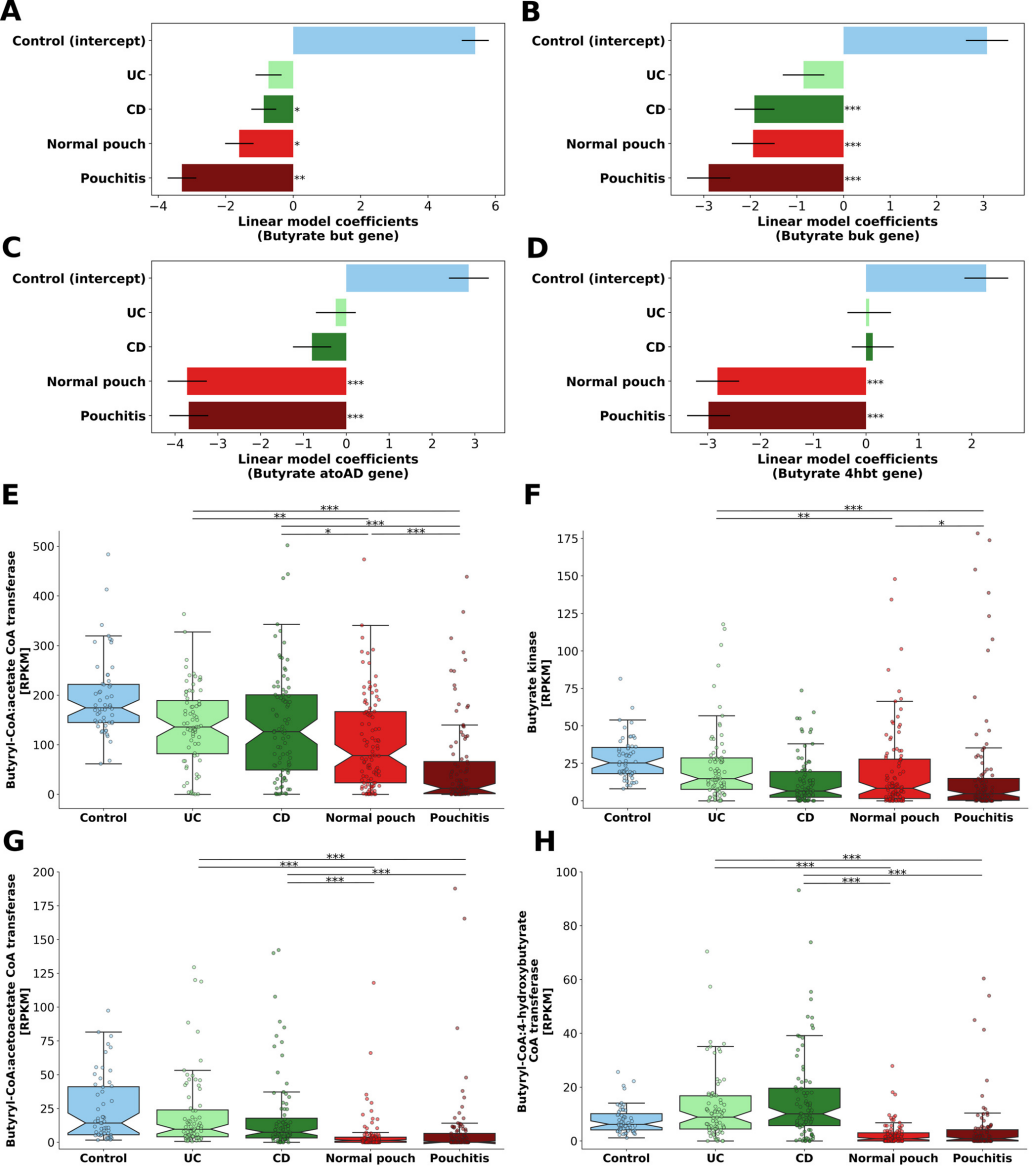
The main pathways of microbial butyrate synthesis in IBD and healthy subjects. Modeling and quantification of the terminal genes (*but*, *buk*, *ato*, and *4hbt*) for corresponding butyrate synthesis pathways in the metagenomes: (A and E) butyryl-CoA:acetate CoA transferase (*but*) pathway; (B and F) butyrate kinase (*buk*)–acetyl-CoA and glutarate pathways; (C and G) butyryl-CoA:acetoacetate CoA transferase (*ato*)–lysine pathway; (D and H) butyryl-CoA:4-hydroxybutyrate CoA transferase (*4hbt*)–4-aminobutyrate pathway. In panels A to D, linear model coefficients (effect sizes) are given for each phenotype for the modeled genes. *, *P* < 0.05; **, *P* < 0.01; ***, *P* < 0.001. In panels E to H, gene abundance is in RPKM units (reads per kilobase per million mapped reads), normalized according to the corresponding median gene length and total-sum scaled to the number of reads in each metagenome. *, *P* < 0.05; **, *P* < 0.01; ***, *P* < 0.001; Kruskal-Wallis, applying Dunn’s multiple correction test. For a list of all pairwise *P* values, see Data Set S1, sheet 12. Box plot whiskers mark observations within the 1.5 interquartile range of the upper and lower quartiles.

We quantified and modeled the terminal genes of the four known butyrate production pathways—namely, *but*, *buk*, *ato*, and *4hbt*—in the metagenomes using an extensive and rigorously curated database for butyrate synthesis genes (31). Overall, patients with CD, normal pouch, and pouchitis had the lowest levels of butyrate synthesis pathways (Fig. 4A to D) (*P* < 0.05). Specifically, according to the *but* gene (Fig. 4A and E), the lowest abundance of these genes was in patients with pouchitis (median RPKM, 12), followed by significantly higher abundance in patients with a normal pouch (median RPKM, 78; Kruskal-Wallis, *P* = 3.5 × 10^−6^). No significant difference in butyrate synthesis genes between CD (median RPKM, 126) and UC (median RPKM, 136) was observed. Analysis of the *buk* homologs (Fig. 4B and F) revealed similar abundances between patients with CD (median RPKM, 6.6) and with normal pouches (median RPKM, 8.4), which were lower than those of patients with UC and healthy subjects (median RPKM, 14.7 and 25.3, respectively; Kruskal-Wallis, *P* < 0.01). Patients with pouchitis had the lowest levels (median RPKM, 4.75). The lysine (*ato*) and 4-aminobutyrate (*4hbt*) pathways were lower in patients with a pouch (Fig. 4C and D) and less abundant in general in all groups (Fig. 4G and H). Patients with a pouch had very low levels of both *ato* and *4hbt* genes (which were absent altogether in many samples), suggesting that the 4-aminobutyrate and lysine pathways for butyrate synthesis are exceptionally low in these patients. Similar trends in all four terminal genes were observed in pouch patients from the American cohort (Fig. S3E to H).

The taxonomic affiliation of butyrate-producing bacteria based on the similarity of the identified *but* and *buk* genes to those of known butyrate producers revealed a dominance of commensal *Firmicutes* such as *F. prausnitzii* and several species of *Roseburia* and *Eubacterium* (Fig. S5). Surprisingly, healthy subjects had a somewhat low relative abundance of some of these butyrate producers compared to subjects with disease phenotypes, which might be a result of the higher bacterial diversity in healthy individuals and consequently a lower “reliance” on a few species for butyrate production. *F. prausnitzii*, Eubacterium hallii, and Subdoligranulum variabile had comparable relative abundances in patients with normal pouches, UC patients, and healthy subjects, while patients with pouchitis and CD had low levels of these taxa. For a complete list of all the identified butyrate producers, see Data Set S1, sheet 5. Notably, a significant negative correlation was observed between the abundance of butyrate synthesis genes and fecal calprotectin (Fig. S6A to D). This was most pronounced for the *but* gene (Spearman *r* = −0.322; *P* = 4.34 × 10^−10^). This suggests that either a reduction in butyrate producers predisposes to intestinal inflammation or butyrate-producing bacteria are more sensitive to intestinal inflammation.

10.1128/mSystems.00984-20.5 FIG S5 Major butyrate producers in the fecal metagenomes of patients with IBD and healthy controls. The relative fraction and taxonomic affiliation of the most abundant butyrate-producing bacteria based on the similarity of the identified *but* (butyryl-CoA:acetate CoA transferase) and *buk* (butyrate kinase) genes to known butyrate producers. Coprococcus comes, Subdoligranulum variabile, and Clostridium perfringens were quantified based on *buk*, and the rest of the species were based on *but*. For a complete list of all the identified butyrate producers, see Data Set S1, sheet 5. Download FIG S5, TIF file, 1.6 MB. Copyright © 2021 Dubinsky et al. 2021 Dubinsky et al. https://creativecommons.org/licenses/by/4.0/ This content is distributed under the terms of the Creative Commons Attribution 4.0 International license.

10.1128/mSystems.00984-20.6 FIG S6 Spearman correlation between the abundance of butyrate synthesis genes (*but*, *buk*, *ato*, and *4hbt*), secondary bile acid formation genes (*baiA*-*baiI* operon), and fecal calprotectin levels in patients with IBD and healthy controls. Spearman correlation for (A to D) four main butyrate synthesis pathways, as in Fig. 4 (*but*, *buk*, *ato*, and *4hbt*), and (E) secondary bile acid (DCA and LCA) formation (*baiA*-*baiI* operon). Lines are linear regression fitted with a 95% confidence interval. Fecal calprotectin measurements were available for 358 of 428 samples. Download FIG S6, TIF file, 1.5 MB. Copyright © 2021 Dubinsky et al. 2021 Dubinsky et al. https://creativecommons.org/licenses/by/4.0/ This content is distributed under the terms of the Creative Commons Attribution 4.0 International license.

As butyrate production may be affected by patients’ dietary habits, especially dietary fiber consumption, we correlated the average daily intake of dietary fiber derived from different food sources with butyrate synthesis genes in a subset of pouch patients (*n* = 52) that had dietary information (food frequency questionnaires [FFQs] obtained at the time of fecal sample collection) (Table 1). The abundances of *but* and *buk* genes were positively correlated with total dietary fiber intake (Spearman *r *= 0.366; *P* = 0.0076) and specifically with intake of fiber from fruit (Spearman *r *= 0.293; *P* = 0.035), vegetables (Spearman *r *= 0.275; *P* = 0.048), and potatoes (Spearman *r *= 0.46; *P* = 6 × 10^−4^). Higher fruit consumption was previously associated with increased abundance of known butyrate-producing species and lower recurrence of pouchitis (32). Nutritional intervention for patients with a pouch, such as the Mediterranean diet, was previously associated with decreased inflammatory markers and later onset of pouchitis and is characterized by a high intake of dietary fiber (33). Our results, therefore, highlight diet as an important factor that may affect butyrate synthesis and provide further support for dietary intervention as a plausible approach to modulate the microbiota for increased butyrate synthesis potential in the pouch.

**TABLE 1:** Spearman correlation between dietary fiber intake and the abundance of butyrate genes (*but* and *buk*) in patients with pouch samples (*n* = 52)

Fiber source	Rho	*P*
Total	0.366	0.0076
Fruits	0.293	0.0346
Vegetables	0.275	0.0479
Potatoes	0.46	6 × 10^−4^
Grains	−0.08	0.5729
Legumes and nuts	0.1	0.4847

Previous studies examining butyrate-producing bacteria, their gene content, or their metabolites in IBD reported various results. A study by Machiels et al. (34) reported no difference in fecal butyrate concentrations between healthy subjects and UC patients, while others reported decreased fecal butyrate in CD and (to a lesser degree) UC patients (35, 36). According to the quantification of *but* gene content, reduced butyrate synthesis was identified in patients with active and inactive CD but only in patients with active UC (37). For patients with a pouch, data about butyrate production are scarce, and butyrate synthesis genes had not been previously analyzed. It was suggested that bacterial dysbiosis in pouchitis causes SCFA deprivation (6), which might be related to its pathogenesis. Fecal levels of SCFA in patients with pouchitis were substantially lower than in patients with a normal pouch (38, 39), which may contribute to the higher pH observed in pouchitis (27). These past observations of butyrate and pH levels in the pouch are in line with our metagenomics-based findings and suggest that a shortage of butyrate (and its producers) is an important contributor to the pathogenesis of pouchitis. This may be clinically relevant, as SCFA enemas were successfully assessed for the treatment of diversion colitis (40) and for UC (41), yet their effect in pouchitis was rarely assessed in small uncontrolled trials and case studies (42). Our findings provide a mechanistic rationale for a reassessment of this therapy in pouchitis.

### Secondary bile acid production potential is low in pouchitis.

The secondary bile acids deoxycholic acid (DCA) and lithocholic acid (LCA) are formed in the colon from primary bile acid metabolism by the microbiota via a multistep 7α-dehydroxylation reaction. Secondary bile acids (DCA and LCA) have extensive effects on host metabolism and play both negative and positive roles in health and disease (43). The level of DCA in bile is thought to be controlled by two major factors: levels and activities of bile acid 7α-dehydroxylating gut microbes and colonic transit time (43). In IBD, impaired fecal bile acid metabolism was observed, with an increase in primary bile acids and a decrease in secondary bile acids (particularly during flares). *In vitro* and *in vivo* experimental models confirmed that DCA and LCA may exert anti-inflammatory effects (44, 45). Enzymes involved in 7α-dehydroxylation are encoded by bile acid-inducible (*bai)* gene clusters present in bacterial genomes from *Ruminococcaceae* and to a lesser extent from *Lachnospiraceae* and *Peptostreptococcaceae* (46). The *bai* gene cluster was found to be present and expressed in the gut microbiomes of most individuals but represented only a small fraction (<1%) of total intestinal bacteria (47).

**FIG 5:**
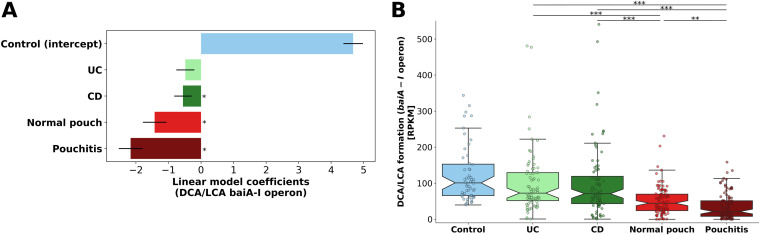
Secondary bile acid (DCA and LCA) formation potential in the metagenomes of patients with IBD and healthy subjects. The *bai* gene cluster (*baiA*-*baiI* operon) was quantified in the metagenomes. (A) Linear model coefficients (effect sizes) are given for each phenotype for the modeled *bai* gene cluster. *, *P* < 0.05. (B) Gene abundance is in RPKM units (see the legend to Fig. 4). *, *P* < 0.05; **, *P* < 0.01; *, *P* < 0.01; Kruskal-Wallis, applying Dunn’s multiple correction test. For a list of all pairwise *P* values, see Data Set S1, sheet 12. Box plot whiskers mark observations within the 1.5 interquartile range of the upper and lower quartiles.

We quantified and modeled the *bai* gene cluster in the metagenomes of IBD and healthy subjects using an extensive and rigorously curated database for genes related to secondary bile acid formation (47). Patients with CD, normal pouch, and pouchitis were significantly associated with a decreased secondary bile acid formation potential (Fig. 5A) (*P* < 0.05). In line with the highest dysbiosis in patients with pouchitis, the *bai* gene cluster abundance was lowest in this group (median RPKM, 21.3) but higher in patients with a normal pouch (Fig. 5B) (median RPKM, 44.6; Kruskal-Wallis, *P* = 0.002). UC and CD patients had comparable levels of *bai* gene abundance (median RPKM, 72.6 and 71, respectively; Kruskal-Wallis, *P* = 0.29), and both were significantly higher than in patients with a pouch (Kruskal-Wallis, *P* < 0.001). A similar pattern in the *bai* gene cluster was observed in pouch patients from the American cohort (Fig. S3I).

Only a few dominant strains in the analyzed metagenomes were identified to carry the *bai* gene clusters (or parts thereof). They belonged to three families of *Firmicutes*: *Lachnospiraceae* (Clostridium scindens), *Peptostreptococcaceae* (Clostridium sordellii and Proteocatella sphenisci), and *Ruminococcaceae* (uncultured *Firmicutes* strain CAG:103 and *Clostridiales* strains UBA4701 and UBA1701). For a complete list of the identified secondary bile acid producers, see Data Set S1, sheet 6. Importantly, the abundance of *bai* gene cluster was significantly negatively correlated with fecal calprotectin (Fig. S6E; Spearman *r* = −0.312; *P* = 1.64 × 10^−9^), implying that DCA- and LCA-producing bacteria are associated with lower levels of intestinal inflammation.

A reduction in secondary bile acid genes in patients with CD or UC (47, 48), as well as a reduction in DCA and LCA metabolites in highly dysbiotic patients with UC and CD, was previously described (13), supporting our current study. The key species carrying *bai* clusters in our metagenomes are concordant with those identified in metagenomes of a healthy population (46). Importantly, one of the prevalent secondary bile acids producers we detected, *C. scindens*, was associated with enhanced resistance to C. difficile infection in a secondary-bile-acid-dependent manner (49). The reduction of secondary-bile-acid-producing bacteria in IBD and particularly in pouchitis might leave these patients more exposed to pathogens or potential pathobionts. Interestingly, patients with an ileal pouch were previously shown to have markedly lower biliary and fecal DCA and LCA levels (compared to healthy controls), while fecal primary bile acid excretion was significantly higher, indicating attenuated bacterial conversion (50). These measurements complement our metagenomic findings and support our hypothesis that a reduction in 7α-dehydroxylation bacterial enzymes leads to a pouch microbiome with lower potential for producing secondary bile acids.

### Enzymes related to protection against oxidative stress are highly prevalent in patients with a pouch.

Previous studies suggested higher oxidative stress in the inflamed gut of patients with UC and CD (9, 25, 51). Here, we aimed to characterize and quantify genes that encode enzymes involved in oxidative stress response (oxygen-detoxifying enzymes) as a proxy for the levels of oxygen tolerance of microbes in the metagenomes of patients with IBD. We used the enzyme profiles annotated with HUMAnN2 to extract and model such oxygen-detoxifying genes. We identified seven enzymes known to be involved in oxidative stress (Data Set S1, sheet 7). However, only glutathione-disulfide reductase (EC 1.8.1.7), which serves to maintain the important antioxidant glutathione in the reduced form (52), was significantly higher in patients with a pouch (mean number of copies per million [CPM], 48.8 and 54.7 in normal pouch and pouchitis, respectively) than in those in UC and CD (mean CPM, 12.9 and 17.2, respectively).

The combined contribution of these seven oxidative stress response enzymes (combined oxidative stress response score) (Fig. S7) was the highest in patients with pouchitis (median RPKM, 401; Kruskal-Wallis, *P* < 0.05), followed by patients with a normal pouch (median RPKM, 298; Kruskal-Wallis, *P* < 0.05), and was significantly lower in the other phenotypes.

10.1128/mSystems.00984-20.7 FIG S7 Combined oxidative stress response genes in the microbiome. The combined contribution of seven oxidative stress response enzymes in metagenomes of patients with IBD and healthy subjects. *, *P* < 0.05; **, *P* < 0.01; ***, *P* < 0.001; Kruskal-Wallis, applying Dunn’s multiple correction test. For a list of all pairwise *P* values, see Data Set S1, sheet 12. Box plot whiskers mark observations within the 1.5 interquartile range of the upper and lower quartiles. Download FIG S7, TIF file, 0.4 MB. Copyright © 2021 Dubinsky et al. 2021 Dubinsky et al. https://creativecommons.org/licenses/by/4.0/ This content is distributed under the terms of the Creative Commons Attribution 4.0 International license.

A clear difference was observed between the species that contributed highly to these antioxidative stress enzymes in pouch and those in nonpouch phenotypes (Fig. S8). In the former, the vast majority consisted of facultative anaerobes such as E. coli and to a lesser degree *Klebsiella* and few species of *Streptococcus* and *Lactobacillus.* In the latter, E. coli contribution was less pronounced and replaced by that of obligate anaerobes such as *Bacteroides* species, several *Clostridiales* species, *A. muciniphila*, Bifidobacterium longum, and Prevotella copri. A similar trend of increased facultative anaerobe abundance was previously observed in patients with CD or UC compared to healthy individuals (25). Our results suggest that microbes that are more oxygen tolerant and encode more oxygen-detoxifying enzymes have a fitness advantage and become more abundant in the highly oxidative environment of the pouch.

10.1128/mSystems.00984-20.8 FIG S8 Bacterial community contribution of enzymes related to protection against oxidative stress in the metagenomes of patients with IBD and healthy subjects. Each stacked bar on the *x* axis represents a sample from the corresponding phenotype (labels above the bars), and the *y* axis shows the log_10_-scaled total community gene abundance (sum-normalized to copies per million [CPM]). Seven such enzymes were identified and quantified: (A) glutamate-cysteine ligase, (B) glutathione synthase, (C) glutathione-disulfide reductase, (D) superoxide dismutase, (E) peroxiredoxin, (F) peptide-methionine sulfoxide reductase, and (G) catalase. The contributions of the top genera are proportionally scaled within the total. “Other” encompasses contributions from additional lower-abundance genera. See the legend at the bottom of the figure for the color codes. The labels over the bars represent the clinical phenotypes: HL, non-IBD; UC, ulcerative colitis; CD, Crohn's disease; NP, normal pouch; and P, pouchitis. Download FIG S8, JPG file, 2.9 MB. Copyright © 2021 Dubinsky et al. 2021 Dubinsky et al. https://creativecommons.org/licenses/by/4.0/ This content is distributed under the terms of the Creative Commons Attribution 4.0 International license.

### Metabolic pathway perturbation in patients with a pouch or CD.

Our next goal was to use an untargeted approach to broadly analyze the functional potential of the microbiome and to identify metabolic pathways (MetaCyc annotations) that might be associated with IBD and with pouch phenotypes in particular. Such hypothesis-free analysis can reveal new functional pathways that may relate to the course of the disease and dysbiosis. We used HUMAnN2 to annotate the metagenomes for metabolic pathways and compared them across disease phenotypes using linear modeling (Data Set S1, sheet 8).

Metabolic pathways for the biosynthesis of the amino acids l-lysine (PWY-5097 and PWY-2942) and l-ornithine (ARGININE-SYN4-PWY) were less abundant (false discovery rate [FDR] *P* < 0.1) in patients with a pouch compared to CD and UC patients. However, the abundances of other amino acid biosynthesis pathways were significantly enriched (FDR *P* < 0.1) in patients with a pouch and to some extent in those with CD: l-alanine (PWY0-1061), phenylalanine (PWY-6628), l-ornithine as an intermediate for l-arginine (GLUTORN-PWY), and l-arginine (ARGSYNBSUB-PWY). These pathways were encoded predominantly by members of the *Enterobacteriaceae* (E. coli, *Klebsiella* sp., and Enterobacter cloacae), *Enterococcus* (e.g., Enterococcus faecium and Enterococcus faecalis) and *Streptococcus* species, which were enriched in CD and in pouch phenotypes in particular (Fig. 2). In addition, we found that patients with CD, normal pouch, and pouchitis had a higher level (mean CPM, 10.3, 20.3, and 37.2, respectively) of molybdenum cofactor synthesis pathway (PWY-6823), encoded by E. coli, which is a crucial cofactor required for anaerobic respiration with nitrate as a terminal electron acceptor during intestinal inflammation (53). Interestingly, none of the above-mentioned amino acid pathways were significantly enriched in UC. It was previously demonstrated that a range of amino acids, including lysine, alanine, and phenylalanine were higher in the fecal metabolomes of patients with active CD and UC compared to healthy controls (54). Our findings are consistent with metagenomic and metabolomic studies of pediatric CD fecal samples that revealed positive associations of bacterial genes for nitrogen metabolism and amino acid synthesis from Proteobacteria (55), which also correlated with higher concentrations of fecal amino acids and their derivatives in CD patients (56). It is still unknown to what degree the higher levels of fecal amino acids are due to inflammation-driven host malabsorption or higher synthesis by the intestinal microbiota.

In addition to biosynthesis pathways, we observed a significant enrichment in the pathway for degradation of the amino acid ornithine (ORNDEG-PWY) in patients with a normal pouch (mean CPM, 16.6) and pouchitis (mean CPM, 30.9) and to a lesser degree in CD patients (mean CPM, 7.3). Moreover, the pathway for degradation of the aromatic organic compound phenylpropanoid (PWY-6690), which can be derived from the putrefaction of proteins, was significantly higher in patients with a normal pouch (mean CPM, 14.5), pouchitis (mean CPM, 27.4), and CD (mean CPM, 10.4). We identified two more pathways for aromatic compound degradation, 3-phenylpropanoate (PWY0-1277) and phenylacetate (PWY0-321), that followed a similar pattern of higher abundance in pouch and to lower extent in CD. These phenolic compounds are important intermediates in bacterial degradation of aromatic molecules. Lastly, galactarate sugar degradation (GALACTARDEG-PWY) was increased in patients with a normal pouch (mean CPM, 20.2), pouchitis (mean CPM, 36.6), and CD (mean CPM, 10.4). The degradation pathways we identified were encoded by *Enterobacteriaceae* species. It was reported that host-mediated galactarate oxidation following antibiotic treatment led to an expansion of Salmonella enterica and potentially other *Enterobacteriaceae* that used this sugar (57). Our results indicate that species of the *Enterobacteriaceae* may benefit from higher protein, aromatic compound, sugar, and galactarate availability, which can explain the relative expansion of this bacterial family in patients with CD (10) and with pouchitis (11, 12).

### Classification models can distinguish between patients with a normal pouch and pouchitis based on species and functions.

**FIG 6:**
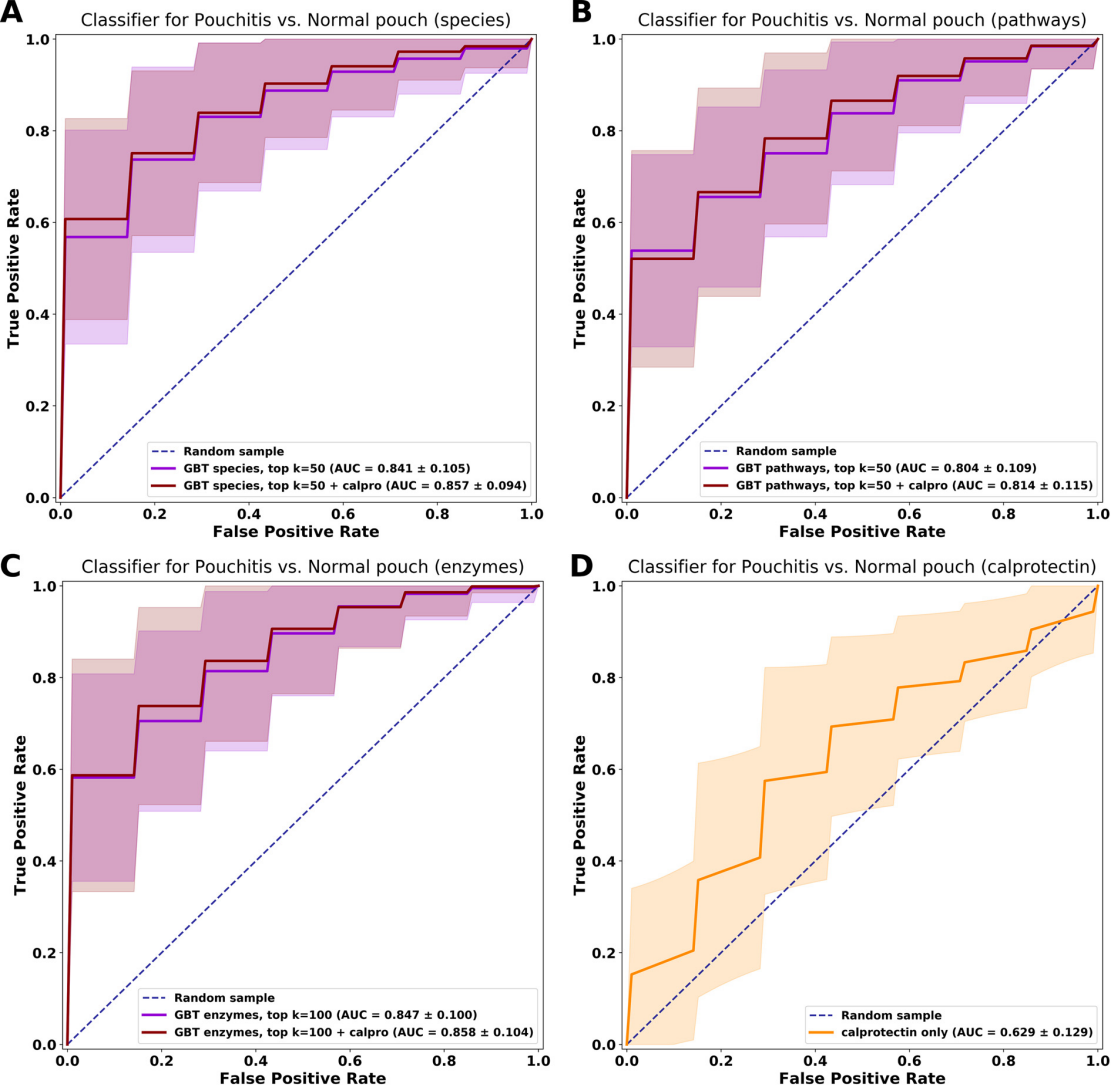
Classification models to distinguish between patients with a normal pouch and pouchitis. We trained and evaluated gradient boosting tree (GBT) classifiers on bacterial, metabolic pathway, and enzyme profiles from the metagenomes of patients with a pouch (discovery cohort) by using 5-fold cross-validation (randomly repeated 100 times). The area under the curve (AUC) (mean ± standard deviation) for each classifier’s performance is presented. (A) Species-based classifier. (B) Metabolic pathway-based classifier. (C) Enzyme-based classifier. (D) Fecal calprotectin-based classifier. For each different classifier (species, pathway, and enzyme based, excepting calprotectin), two different models were built, with top-scoring features based on importance scores with and without fecal calprotectin as an additional predictor.

We observed multiple differences in species and functional genes between patients with a normal pouch and those with pouchitis, yet no bacterial feature on its own was highly discriminative. We, therefore, attempted to use species, metabolic pathway, and enzyme profiles in order to build classifiers to distinguish between these pouch phenotypes in the discovery cohort. We used a machine learning algorithm of gradient boosting trees (GBT) to train classifiers and evaluated its performance using 5-fold cross-validation. We designed our models based on the averaging of longitudinal data per subject, a classifier approach that was shown to be superior to the use of a single sample per subject (58). Our pouch cohort (discovery) consisted of 208 samples from 69 patients (*n* = 35 for normal pouch, *n* = 34 for pouchitis). To improve the model’s predictive power, we used the feature importance scores that the algorithm assigns to each feature used during training; therefore, features with the highest scores are the most informative for correct classification. A GBT classifier trained on the 50 top-scoring features (species) achieved a mean area under the curve (AUC) of 0.841 ± 0.105 (Fig. 6A). Slightly improved outcomes were noticed when fecal calprotectin was incorporated into the model as a predictor (AUC = 0.857 ± 0.094). Next, we built GBT models with metabolic pathways (MetaCyc) and microbial enzymes (ECs) as features. The pathway-based GBT model with the 50 top-scoring features achieved an AUC of 0.804 ± 0.109, a worse performance than the species-based classifier, while the addition of calprotectin only slightly improved the model (AUC = 0.814 ± 0.115). Interestingly, the GBT models trained on the top-100-ranking bacterial enzymes achieved classification scores (without and with calprotectin, AUC = 0.847 ± 0.1 and AUC = 0.858 ± 0.104, respectively) comparable to those of the species-based models, despite having a larger number of features (Fig. 6C). Furthermore, as the sole predictive feature, fecal calprotectin achieved a poor AUC of 0.625 ± 0.129 (Fig. 6D) in distinguishing normal pouch from pouchitis, emphasizing that intestinal inflammation alone cannot explain the observed differences between pouchitis and normal pouch phenotypes.

Next, we validated our model on 20 pouch patients from our center (not part of the discovery cohort) and on an American cohort of 15 patients with a pouch (17). The species- and enzyme-based models trained on the discovery cohort and tested on the validation cohort performed well, achieving AUC of 0.812 and 0.796, respectively (Fig. S9A and B). In terms of accuracy, the species model correctly classified 25/35 patients (71.4%), while the enzymes model correctly classified 27/35 patients (77.1%), suggesting that our classifier can generalize fairly well on unseen data (Fig. S9C and D).

10.1128/mSystems.00984-20.9 FIG S9 Classification models testing on a validation cohort of 20 pouch patients from our center (independent from the discovery cohort) and on an American cohort of 15 patients with a pouch (17). (A and C) Area under the curve (AUC) performance measure of species and enzyme classifiers, respectively, trained on the discovery cohort and tested on the validation cohort. (B and D) Confusion matrix evaluation of the species and enzyme classifiers, respectively, comparing the number of correctly and incorrectly classified samples. Download FIG S9, TIF file, 0.8 MB. Copyright © 2021 Dubinsky et al. 2021 Dubinsky et al. https://creativecommons.org/licenses/by/4.0/ This content is distributed under the terms of the Creative Commons Attribution 4.0 International license.

**FIG 7:**
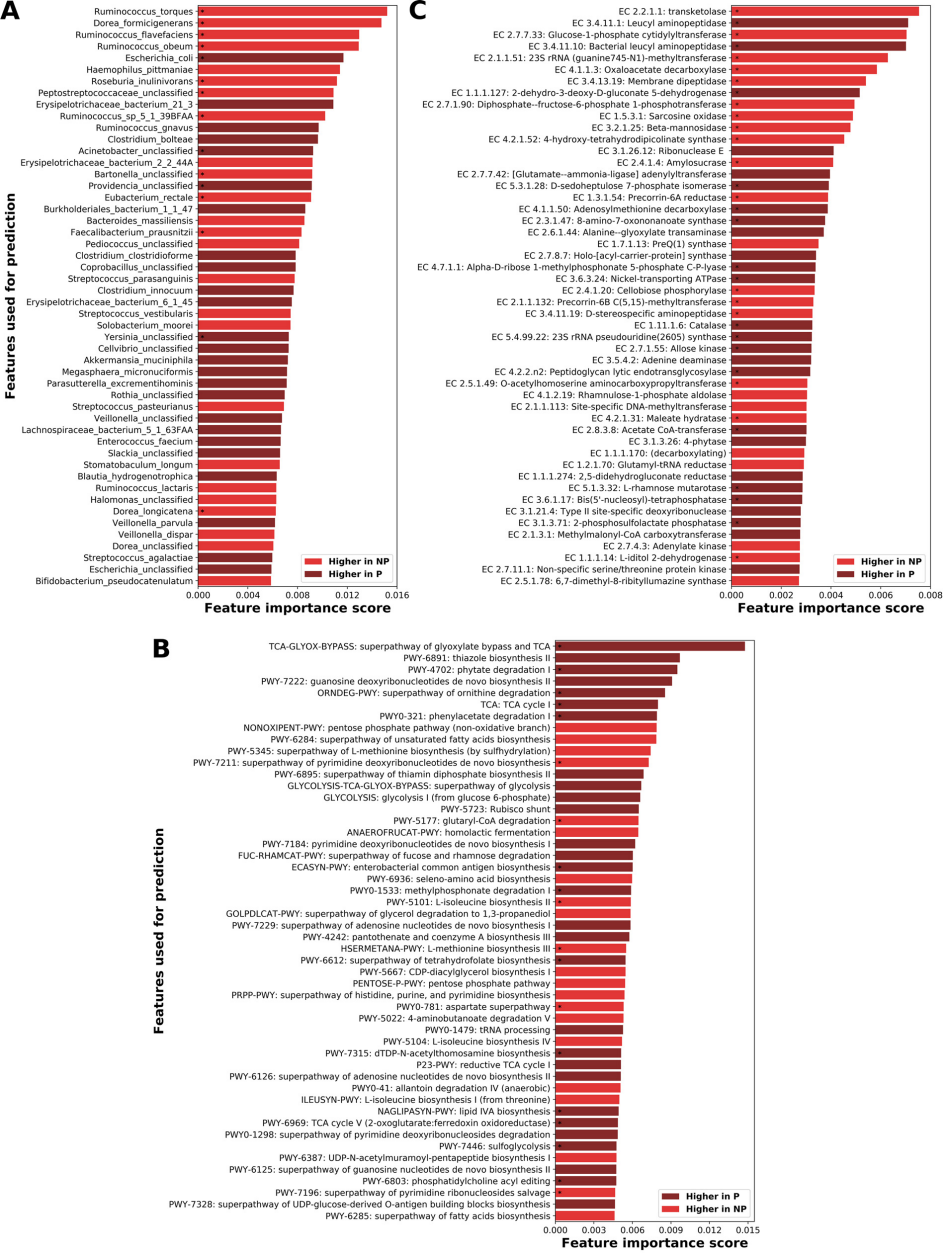
Highest-scoring microbiome features identified by the classification models as the most informative for distinguishing between patients with a normal pouch and pouchitis and thus possibly predictive of pouchitis. The feature importance scores (averaged across 5-fold cross-validation) for (A) species, (B) metabolic pathways, and (C) enzymes. The lighter red and darker red denote features higher in normal pouch (NP) and pouchitis (P) groups, respectively. For a list of all the importance scores for the features of each model, see Data Set S1, sheets 9 to 11.

We analyzed the top-ranking features from each model in order to identify which species and functions are the most discriminatory between the pouch phenotypes and thus might serve as potential predictors of pouchitis. Among the 50 species with the highest importance score, 15 were significantly different (Mann-Whitney, FDR *P* < 0.1) between normal pouch and pouchitis (Fig. 7A; Data Set S1, sheet 9). Commensal *Ruminococcaceae* (Ruminococcus obeum and Ruminococcus flavefaciens) and *Lachnospiraceae* (Dorea formicigenerans and Dorea longicatena) taxa and beneficial species such as *F. prausnitzii*, E. rectale, and Roseburia inulinivorans were more abundant in the normal pouch. Taxa that were enriched in the pouchitis group and might be inflammation associated were E. coli, an unclassified *Providencia* species, and *Yersinia* sp. (all members of the *Enterobacteriaceae*) and an unclassified Acinetobacter sp. (*Moraxellaceae*). Interestingly, *Providencia* species have been implicated in diarrhea, urinary tract infections, and sepsis (59), and members of the genus *Yersinia*, which includes enteric human pathogens, were found in ∼7.7% of ileal biopsy specimens of both CD patients and controls (60). In a cohort of pediatric CD patients from a non-Western population, levels of Acinetobacter organisms were found to be significantly higher than in controls (61). We noted that three of the above-mentioned mucolytic bacteria appeared in the top-ranking species: *R. torques* was higher in normal pouch (Mann-Whitney, FDR *P* < 0.1), while *R. gnavus* and *A. muciniphila* were higher in pouchitis (not statistically significant).

Metabolic pathways with a high-ranking score in differentiating patients with a pouch (Fig. 7B; Data Set S1, sheet 10) included some general core metabolic pathways present in many organisms and thus merely reflected differences in species diversity (tricarboxylic acid [TCA] cycle, pentose phosphate pathway, and nucleoside and nucleotide biosynthesis). Many of the pathways that were enriched in pouchitis were encoded primarily by E. coli and several other *Enterobacteriaceae* that were more abundant in these patients. Potentially important degradation pathways of phytate, phenylacetate, ornithine, methylphosphonate, and sulfoquinovose were higher in pouchitis (Mann-Whitney, FDR *P* < 0.05). In addition, the pathway for biosynthesis of the lipid A (NAGLIPASYN-PWY) component of lipopolysaccharide (LPS) was significantly enriched in pouchitis. Lipopolysaccharide is a potent activator of innate immunity via Toll-like receptor 4, and patients with CD and UC, especially active ones, have been found to have increased serum levels of LPS (62). On the other hand, the biosynthesis of l-isoleucine and l-methionine was higher in patients with a normal pouch (Mann-Whitney, FDR *P* < 0.05). We also explored the enzymes that were highly ranked by the model (Fig. 7C; Data Set S1, sheet 11) and were differentially abundant between the phenotypes (Mann-Whitney, FDR *P* < 0.05). Levels of genes for two putative aminopeptidases (EC 3.4.11.1 and EC 3.4.11.10) as well as a gene for rhamnose sugar degradation (l-rhamnose mutarotase) were increased in pouchitis. Additional enzymes that were higher in pouchitis included adenosylmethionine decarboxylase, which is involved in the biosynthesis of spermidine, a polyamine that may play a role in survival and virulence of human bacterial pathogens (63), and d-sedoheptulose 7-phosphate isomerase, which is involved in the biosynthesis of d-mannoheptose, an LPS component. Notably, catalase (EC 1.11.1.6), which detoxifies hydrogen peroxide, was significantly higher in pouchitis, indicating the presence of bacteria able to sustain higher levels of oxidative stress (facultative aerobes). In the normal pouch, genes related to vitamin B_12_ biosynthesis (EC 1.3.1.54, EC 2.1.1.132, and EC 2.1.1.133), starch, sucrose, and galactose metabolism (EC 2.4.1.4 and EC 3.2.1.10), sorbitol sugar degradation (EC 1.1.1.14), and homocysteine biosynthesis (EC 2.5.1.49) were enriched.

In summary, some of the enzymatic functions enriched in pouchitis result from the enrichment in Gram-negative bacteria (LPS) and in facultative aerobes that may benefit from intestinal inflammation. In contrast, normal pouch often has a broader diversity in metabolic capacities that accompanies the higher species diversity (Fig. 1D).

### Conclusions.

In this study, we explored species and functions in the microbiomes of a well-phenotyped cohort of patients with an ileal-anal pouch. We identified several metabolic pathways and enzymes that were altered in patients with a pouch and in CD. Most notably, patients with pouchitis had lower levels of genes for the synthesis of butyrate and secondary bile acids and harbored more facultative anaerobic bacteria, suggesting high oxidative stress during pouch inflammation. No excessive bacterial mucus utilization or degradation in IBD was found, but rather, a change in the mucolytic bacterial community composition, highlighted by an increased fraction of *R. gnavus* strains encoding fucosidases in patients with pouchitis, was seen. Patients with a pouch that later develop pouchitis are often considered a model for *de novo* inflammation in CD (14). Here, we show that the normal pouch is already dysbiotic (function- and species-wise), and based on multiple pathways, such as higher amino acid biosynthesis and degradation of aromatic compounds and sugars, encoded primarily by members of *Enterobacteriaceae*, pouch phenotypes are more similar to CD than to UC. Pouchitis thus represents a more extreme case of the various trends that constitute the dysbiosis observed in CD. Our machine learning models identified several species and genes that were predictive of pouchitis. Our analysis was limited by a relatively small number of patients and reliance on publicly available metagenomes from other cohorts for the nonpouch phenotypes, which introduces some country-specific biases. Nevertheless, we validated our analysis and models using a validation cohort and achieved a reasonably good classification. Our findings also delineate microbial and functional factors that underlie the pathogenesis of pouchitis and may suggest pathways that could be targeted for intervention in combination with dietary changes, such as higher intake of fiber, to attenuate small intestinal inflammation in pouchitis and CD. Future studies should generate higher-resolution clinical and nutritional data combined with microbial-based biomarkers which might be integrated into diagnostic tools to assist clinical decision-making.

## MATERIALS AND METHODS

### Study cohort description and metadata characteristics.

Patients after pouch surgery were recruited at a dedicated pouch clinic in a tertiary IBD referral center in Israel (Rabin Medical Center [RMC]). The study was approved by the local institutional review board (0298–17) and the National Institutes of Health (NCT01266538). Demographic and clinical data were obtained during clinic visits. After providing written informed consent, patients were followed prospectively, and clinic visits, including fecal sample collection, were scheduled every 3 months or when patients had an episode of pouchitis. Fecal samples were collected in sterile cups and immediately frozen at −80°C until processing. Fecal calprotectin levels were measured. Pouch disease behavior (phenotype) was defined as normal pouch, acute/recurrent-acute pouchitis, chronic pouchitis, or Crohn’s-like disease of the pouch (CLDP); see “Pouch disease behavior (pouch phenotype)” below for a detailed description. Disease activity was defined based on global physician assessment and on the clinical subscore of the pouchitis disease activity index (64).

For the exploratory analysis done in this study, the patients were divided into two groups (discovery cohort): normal pouch (*n* = 35; 103 fecal samples) and pouchitis (*n* = 34; 105 fecal samples) (Data Set S1, sheet 1). The normal pouch group included samples from patients with a normal pouch (with a UC or familial adenomatous polyposis [FAP] background), while the pouchitis group included samples from patients with chronic pouchitis and CLDP. For the validation cohort used for testing the machine learning models (see “Machine learning classifiers” below), we included 20 pouch patients from our center (not part of the discovery cohort) and 15 pouch patients from the American cohort (17) (Data Set S1, sheet 2).

We excluded samples collected during or within 1 month of antibiotic treatment. Of the pouch samples in our data set, 84% were from patients who had been off antibiotics for 180 days or longer and the rest were from patients who had been off antibiotics for 1 to 6 months (Data Set S1, sheet 1). While patients with pouchitis tend to have a history of high antibiotic use, after 1 month off antibiotics, the microbiome diversity is significantly increased, and following 6 months postantibiotics, it recovers to pretreatment levels (12). Nevertheless, to account for any remaining confounding antibiotic effects, we also included antibiotic use (1 month or 6 months without antibiotics) as a covariate in the linear models.

Nonpouch IBD (patients with UC and CD) and healthy-adult metagenomes were obtained from previously published, publicly available data (18) and are part of the PRISM, LifeLines DEEP, and NLIBD cohorts. We analyzed these metagenomes with the same bioinformatic pipeline as the pouch metagenomes as described below, starting the analysis from raw reads.

### Pouch disease behavior (pouch phenotype).

Pouch disease behavior was defined as normal, acute/recurrent-acute pouchitis, chronic pouchitis, or CLDP, as previously defined (65). Briefly, a normal pouch was defined as one with no symptoms of pouchitis during the past 2 years and no antibiotic therapy. Acute/recurrent-acute pouchitis was defined as a flare of pouchitis responding to a short course (usually 2 weeks) of antibiotic therapy, or up to 4 flares/year, respectively. Chronic pouchitis was defined as >4 pouchitis flares/year or administration of antibiotics or IBD-specific anti-inflammatory therapy for more than 1 month. CLDP was defined as pouch-perianal disease, pouch strictures, or long segments of proximal small intestinal inflammation. Patients undergoing pouch surgery due to familial adenomatous polyposis (FAP) were recruited as well and had a normal pouch throughout follow-up.

### Dietary information based on food frequency questionnaires.

We obtained dietary-fiber information for a subset of 52 samples of patients with a pouch (*n* = 36). Intake of food groups and nutrients was assessed using food frequency questionnaires (FFQs) adapted to the Israeli population and administered in the MABAT Israeli nutrition and public health governmental study. For each of the 106 food items, a commonly used portion size was specified. Patients were asked how often on average they consumed each food item during the 6-month period prior to each visit, and daily intake of nutrients was calculated by multiplying the frequency of each food item by the nutrient content based on the Israeli TZAMERET food database and the United States Department of Agriculture food database (66).

### DNA extraction and shotgun metagenomic sequencing of pouch samples.

DNA was extracted from fecal samples as described in reference 11. Genomic libraries were prepared with the Nextera XT library preparation kit using approximately 1 ng of total DNA per sample (DNA concentrations verified by Qubit fluorometry). Metagenomic sequencing was done using Illumina (San Diego, CA) NextSeq500 paired-end (2 × 150-bp reads) at DNA Services Facility, University of Illinois, Chicago, IL. Sequencing reads were quality filtered with Trimmomatic v0.36 (67) using default parameters, thus removing low-quality and short reads and low-quality bases and clipping Illumina adaptors. Human host DNA reads were removed by mapping metagenomic reads against the GRCh38 human genome (build 38) with Bowtie2 version 2.2.9 (68) in –very-sensitive-local mode. After read trimming and human read removal, the mean sequence depth (± standard deviation) was 0.93 ± 0.3 Gbp (6.22 ± 2 million reads) per sample.

### Taxonomic and functional profiling of the metagenomic data sets.

Taxonomic profiling was performed using MetaPhlAn2 classifier v2.6.0 (69), which classifies metagenomic reads by mapping to a database of clade-specific marker genes. MetaPhlAn2 was run with the following parameters changed from default: –tax_lev set to “s” (classify taxonomy to species level) and –ignore_virus and –ignore_eukaryotes to ignore viral and eukaryote reads, respectively. Species relative abundance tables from all samples were merged. Species present in less than 5% of the samples and at less than 0.1% relative abundance in any individual sample were removed. For all downstream statistical analysis, we used the bacterial species that are common to the pouch and nonpouch metagenomes (species intersection). This procedure can minimize potential confounders, such as species that appear solely in one data set due to country-specific differences. The only exception was for the Shannon diversity index calculation, where all taxa (union) from both data sets were used in order to obtain a more complete and accurate diversity trend.

Functional profiling was performed with HUMAnN2 v0.11.1 (70). Briefly, HUMAnN2 first maps metagenomic reads (nucleotide level with Bowtie2) against functionally annotated pangenomes of the species identified during taxonomic profiling. Reads that do not map to any identified pangenome are subjected to a translated search with DIAMOND (71) against the UniRef90 protein database, and hits are weighted according to alignment quality, sequence length, and coverage. Gene-level outputs are produced in reads per kilobase units and stratified according to known/unclassified microbiome contributions. Per-sample gene abundances were total-sum scaled and normalized (to account for the number of reads in each metagenome) to copies per million (CPM). The obtained gene abundances were regrouped to MetaCyc metabolic pathways and enzymes (using the Enzyme Commission number [EC]).

### Screening the metagenomes for key bacterial functions with custom curated databases.

For the identification and quantification of butyrate synthesis pathways, an extensive database from reference 31 was used, which encompassed hidden Markov models (HMM) on full-length proteins, considering the entire taxonomic diversity of butyrate producers, which contained 1,716 genomes. A modified version of this database was used so that only four terminal enzymes in the main pathways known for butyrate production from each of the genomes were considered. In both the acetyl-CoA and glutarate pathways, the terminal enzymes are butyryl-CoA:acetate CoA transferase (*but*) and butyrate kinase (*buk*). In the 4-aminobutyrate pathway, the terminal enzyme is butyryl-CoA:4-hydroxybutyrate CoA transferase (*4hbt*), and in the lysine pathway, the terminal enzyme is butyryl-CoA:acetoacetate CoA transferase (*ato*). For the analysis of microbiome-derived secondary bile acids, we used the database from reference 47, which was built on the full-length HMM for the *bai* gene cluster (*baiA*-*baiI* operon), which consists of *baiA*, *baiB*, *baiCD*, *baiE*, *baiF*, *baiG*, *baiH*, and *baiI* genes, obtained from all genomes available in PATRIC.

Quality-filtered metagenomic reads were used as a query in a translated nucleotide search (BLASTX using DIAMOND v0.9.24.125 [71]) against the databases described above. Only the top hit per read was reported (–max-target-seqs 1), and the minimum E value threshold was set to 1 × 10^−5^ (–evalue). For butyrate synthesis genes, only reads with sequence similarity matches of ≥90% to a reference and an amino acid alignment length of ≥25 were retained and counted. For bacterial secondary bile acid production genes, the sequence similarity cutoff was lowered to ≥70% due to a less comprehensive reference database. Read counts for each of the key functions analyzed (butyrate and secondary bile acids genes) were normalized to RPKM units (reads per kilobase per million mapped reads), i.e., normalized according to the corresponding median gene length and total-sum scaled according to the number of reads in each metagenome. The key functional genes were assigned to bacterial taxa according to the taxonomic identity of the respective gene entries in the database.

### Mucin degradation glycoside hydrolase analysis.

For the analysis of glycoside hydrolases (GH) that are involved in mucin degradation, only GH that were functionally characterized by *in vitro* activity or transcriptomic assays (72) were considered. Thus, the following enzymes from HUMAnN2 EC4 annotations were considered mucin-related GH: protein *O*-GlcNAcase (EC 3.2.1.169), exo-alpha-sialidase (EC 3.2.1.18), β-galactosidase (EC 3.2.1.23), α-*N*-acetylgalactosaminidase (EC 3.2.1.49), α-*N*-acetylglucosaminidase (EC 3.2.1.50), α-l-fucosidase (EC 3.2.1.51), and β-*N*-acetylhexosaminidase (EC 3.2.1.52).

To analyze the specific GH of Ruminococcus gnavus, GH29 (α-l-fucosidase), GH95 (glycoside hydrolase family 95), and GH33 (exo-alpha-sialidase) protein sequences of Ruminococcus gnavus ATCC 29149 strain JCM6515 were downloaded from the Carbohydrate-Active enZYmes (CAZY) database (73). Three single-copy housekeeping genes (DNA gyrase subunit A [*gyrA*], recombinase A [*recA*], and 50S ribosomal protein L2 [*rplB*]) of the same *R. gnavus* strain were downloaded as well. These GH and the housekeeping genes were used to build a reference database for a similarity search, as mentioned above. For GH, sequence similarity matches of ≥90% and ≥25 amino acids, alignment length was used, and for housekeeping genes, sequence similarity matches were increased to ≥97% to count only closely related strains. Read counts were normalized by the corresponding gene length, and the percentage of *R. gnavus* strains encoding GH29, GH95, and GH33 were calculated by dividing each GH by the mean abundance of *gyrA*, *recA*, and *rplB*. As *gyrA* recruited the highest number of reads even after gene length normalization, we used only *gyrA* for the final calculation. Samples with fewer than 5 *gyrA* reads of *R. gnavus* were removed from the analysis.

### Statistical analysis.

The Kruskal-Wallis H test with Dunn’s test for multiple-comparison correction (pairwise tests between phenotypes) was performed using kruskal.test and dunn.test functions in the R package dunn.test. Alpha-diversity (within samples) was calculated with the Shannon diversity index using the diversity function in the R package vegan. Beta-diversity (between samples) was calculated with Bray-Curtis dissimilarity (the vegdist function in the R package vegan) and visualized with the principal-coordinate analysis function cmdscale (classical multidimensional scaling) in R. Spearman rank correlation between the first axis of microbiome variation, Shannon diversity, and key functional genes and fecal calprotectin was performed using the cor.test function in R.

To calculate the proportion of variance explained by each tested variable on Bray-Curtis dissimilarity (for species, pathways, and enzymes), permutational multivariate analysis of variance (PERMANOVA) was performed using the adonis function in the R package vegan. The variables included disease phenotype, age, antibiotic use, fecal calprotectin, gender, disease activity, pouch phenotype, and intersubject variation (the last four variables were available only for the cohort of patients with a pouch and were fitted separately). The variance explained by each variable was calculated independently of other variables to avoid issues related to variable ordering.

To test for differently abundant microbiome features (species, enzyme, and pathway profiles) in different IBD phenotypes, generalized linear mixed-effects models from the R package MaAsLin2 v0.99.12 (http://huttenhower.sph.harvard.edu/maaslin) were used. Bacterial taxonomy and gene relative abundance data were log-transformed, and additive smoothing of minimum nonzero values was applied to zero values on a per-sample basis. The transformed abundances were modeled as a function of IBD phenotype (with healthy subjects as the reference point) while adjusting for age and antibiotic use (fixed effects). For antibiotic use, a time window of 1 month (recent use) or 6 months off antibiotics was used. To account for the longitudinal data set from pouch patients (repeated measurements), individual patients from whom a set of samples were derived were specified as a random effect (patient ID). To account for potential confounders due to the combination of several cohorts of patients, the cohort from which each sample was derived was specified as another random effect (cohort ID). All reported *P* values were adjusted for multiple-hypothesis testing using the Benjamini-Hochberg method. For differential abundance analysis of key bacterial genes (*but*, *buk*, *atoAD*, *4hbt*, and the *bai* gene cluster) in IBD phenotypes, a linear mixed-effects model was used with the same fixed and random effects as described above, with the lmer function from the R package lme4.

### Machine learning classifiers.

Profiles of bacterial species, metabolic pathways, and enzymes composition were used to build classification models to distinguish between pouch phenotypes (normal pouch versus pouchitis). The machine-learning algorithm of gradient boosting trees (GBT) was used, as implemented in the Python package XGBoost (eXtreme Gradient Boosting [74]) with the XGBClassifier function. The best hyperparameters for each model were tuned empirically using a grid search matrix (GridSearchCV function in the Python scikit-learn package). Briefly, the learning rate was 0.1, the number of boosted trees was 250 (n_estimators), each tree was constructed randomly with 50% of the features for species and pathway models and with 10% for the enzyme model (colsample_bytree), trees were randomly trained on 50% of the samples (subsample), and maximum tree depth was 3 (max_depth). The following regularization parameters were used to reduce model complexity and overfitting: gamma = 0.1 (minimum loss reduction required to make a further partition on a leaf node), reg_alpha = 0.01 (L1 lasso), and reg_lambda = 0.5 (L2 ridge). The rest of the hyperparameters were left at the default settings. The GBT classifiers were based on the averaging of longitudinal microbiome data per subject (discovery cohorts of *n* = 35 for normal pouch and *n* = 34 for pouchitis classes). The classifiers were trained and evaluated on the discovery cohort by using 5-fold cross-validation (randomly repeated 100 times) with StratifiedKFold function (scikit-learn package), which preserves the same distribution of classes in each fold as in the complete training set. The classifiers were tested on the validation cohort, which consisted of 20 pouch patients from our center (not part of the discovery cohort), and on an American cohort of 15 patients with a pouch (17). The model performance was assessed by calculation area under the curve (AUC), sensitivity, specificity, and accuracy metrics.

To improve the model’s predictive power (considering only the most informative features) and reduce complexity (reducing the feature space by removing uninformative and correlated features), feature importance scores were computed using the internal XGBoost function (feature_importances_), which assigns a score to each feature used in the decision tree during model training. The average feature importance scores for each model (species, pathways, and enzymes) were calculated by considering the mean score of each feature for each fold across the repeated (×100) 5-fold cross-validation.

### Data sources.

This study used our previously published sequence data from PRJNA524170. Metagenomes from patients with UC, patients with CD, and healthy controls were obtained from PRJNA400072. Additional pouch metagenomes from the American cohort were obtained from PRJNA600269.

### Data availability.

All the metagenomic sequence data generated and used in this study (patients with a pouch) have been deposited in NCBI SRA and are available under BioProject number PRJNA637365. Analysis scripts with code for the machine learning classifiers are available in the GitHub repository (https://github.com/VadimDu/pouchitis_classifiers).
