# Supplementary figures and images for: Dysbiosis in Metabolic Genes of the Gut Microbiomes of Patients with an Ileo-anal Pouch Resembles That Observed in Crohn's Disease
Source: mSystems. 2021 Mar 2;6(2):e00984-20. doi: 10.1128/mSystems.00984-20 (PMC8546988; doi:10.1128/mSystems.00984-20)

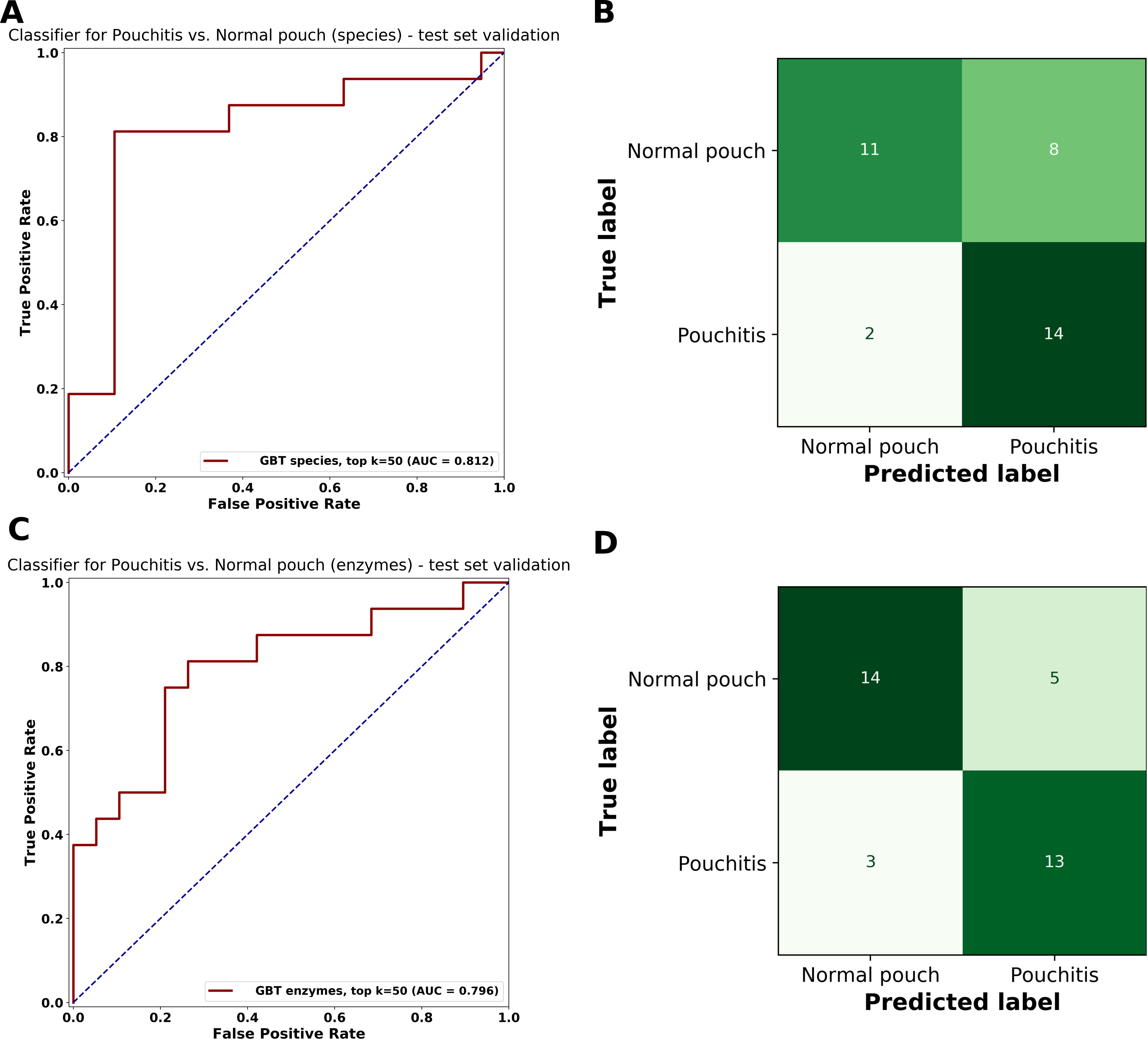

Supplement: FIG S1 [file msystems.00984-20-sf001.tif]

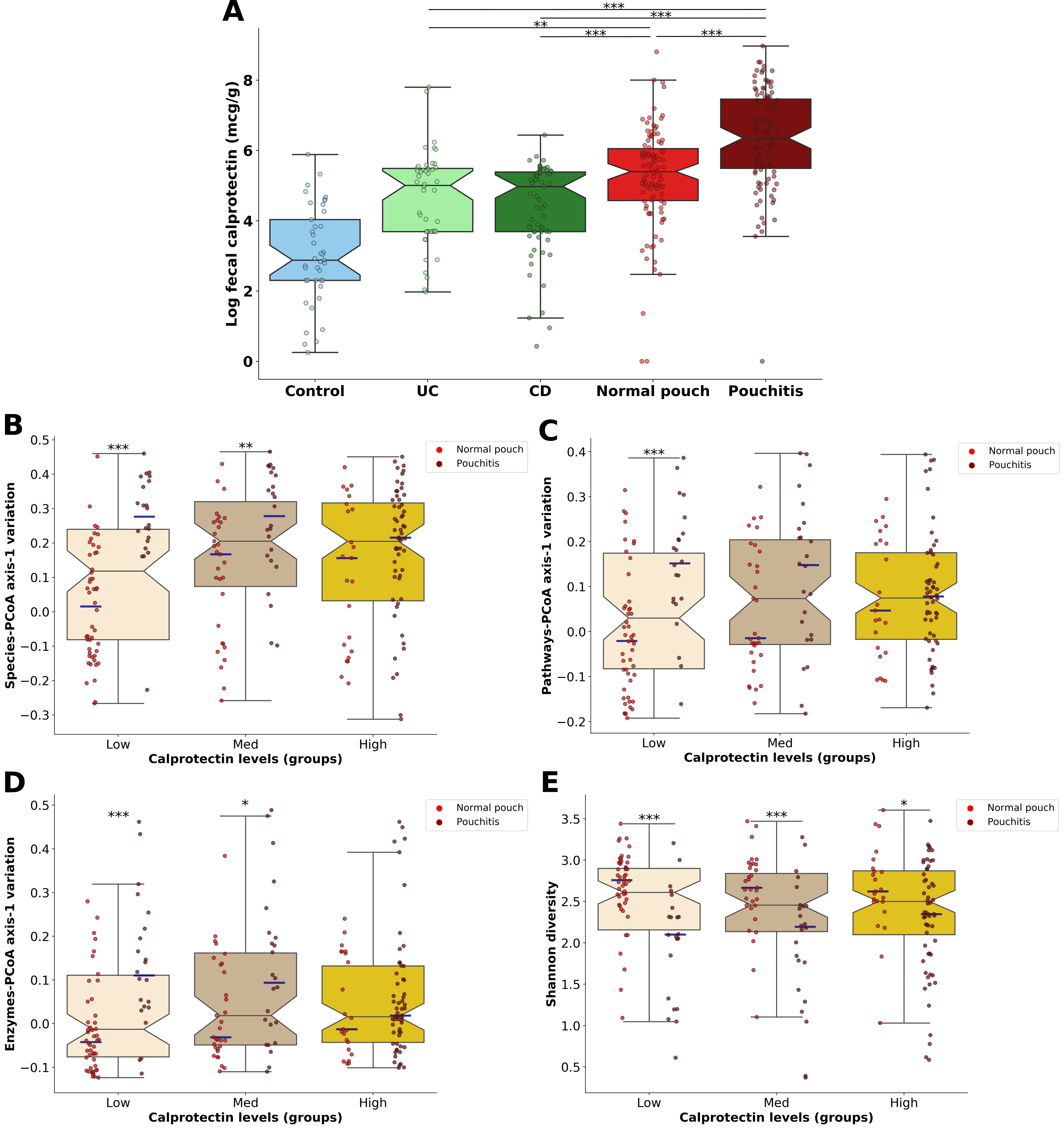

Supplement: FIG S2 [file msystems.00984-20-sf002.tif]

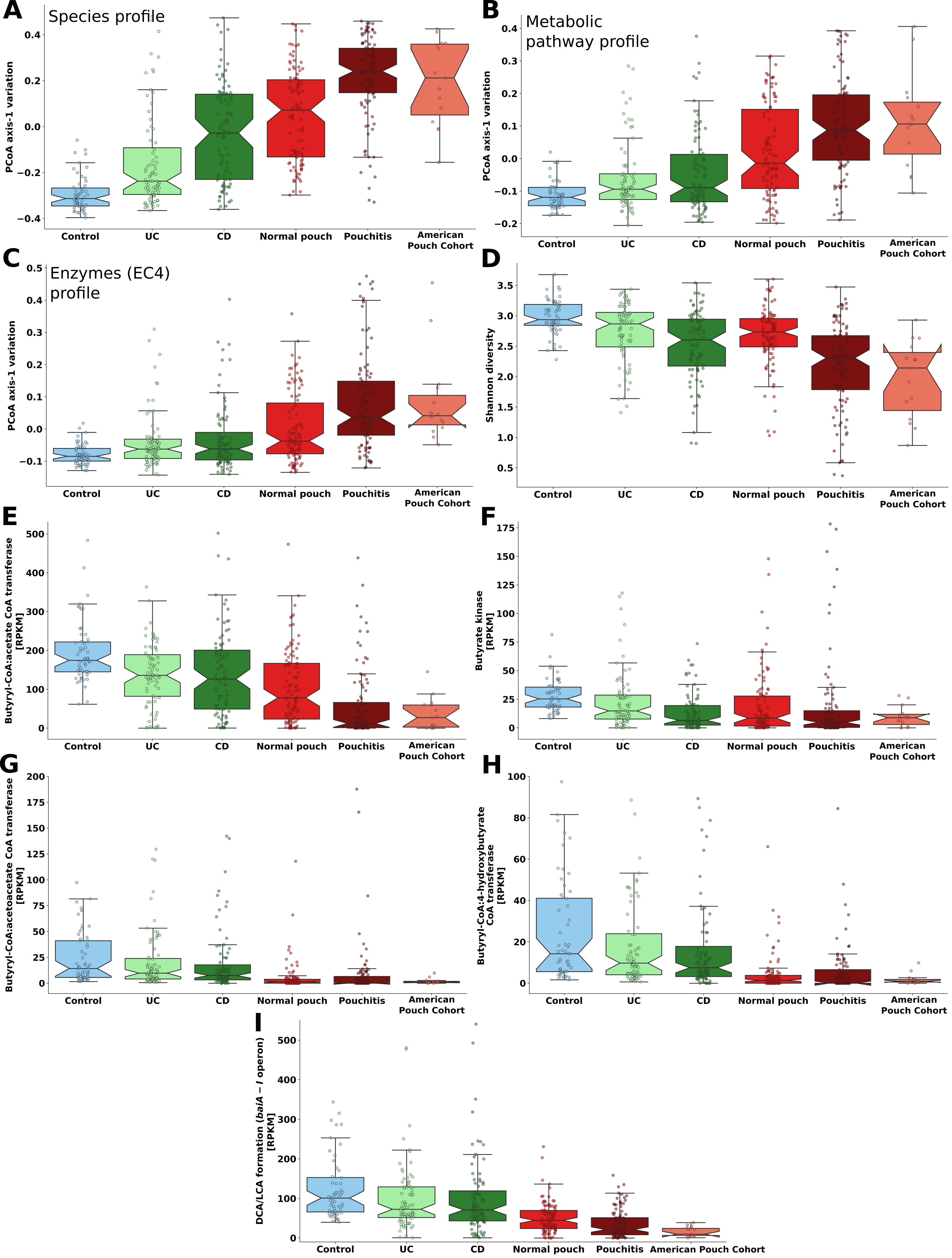

Supplement: FIG S3 [file msystems.00984-20-sf003.tif]

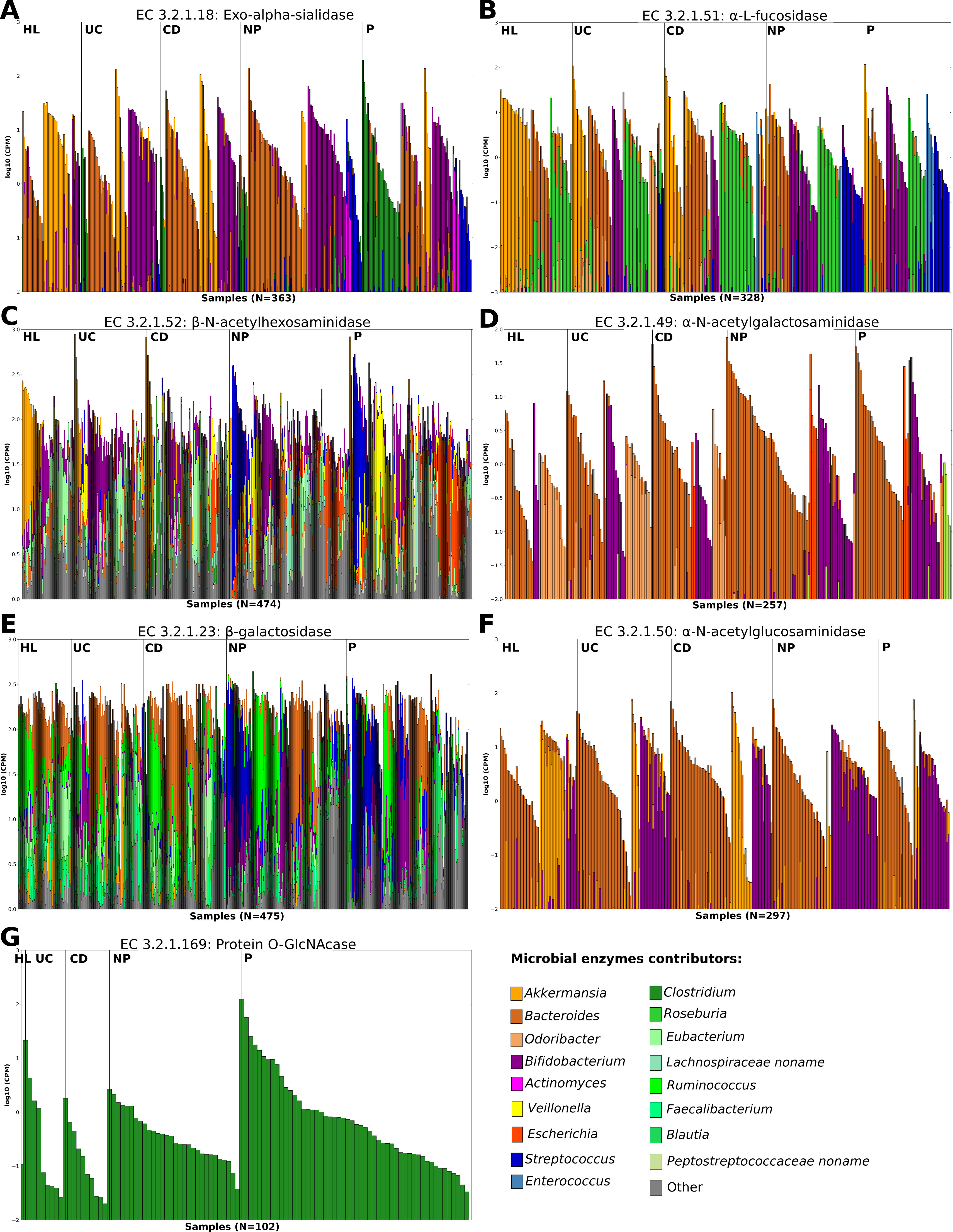

Supplement: FIG S4 [file msystems.00984-20-sf004.jpg]

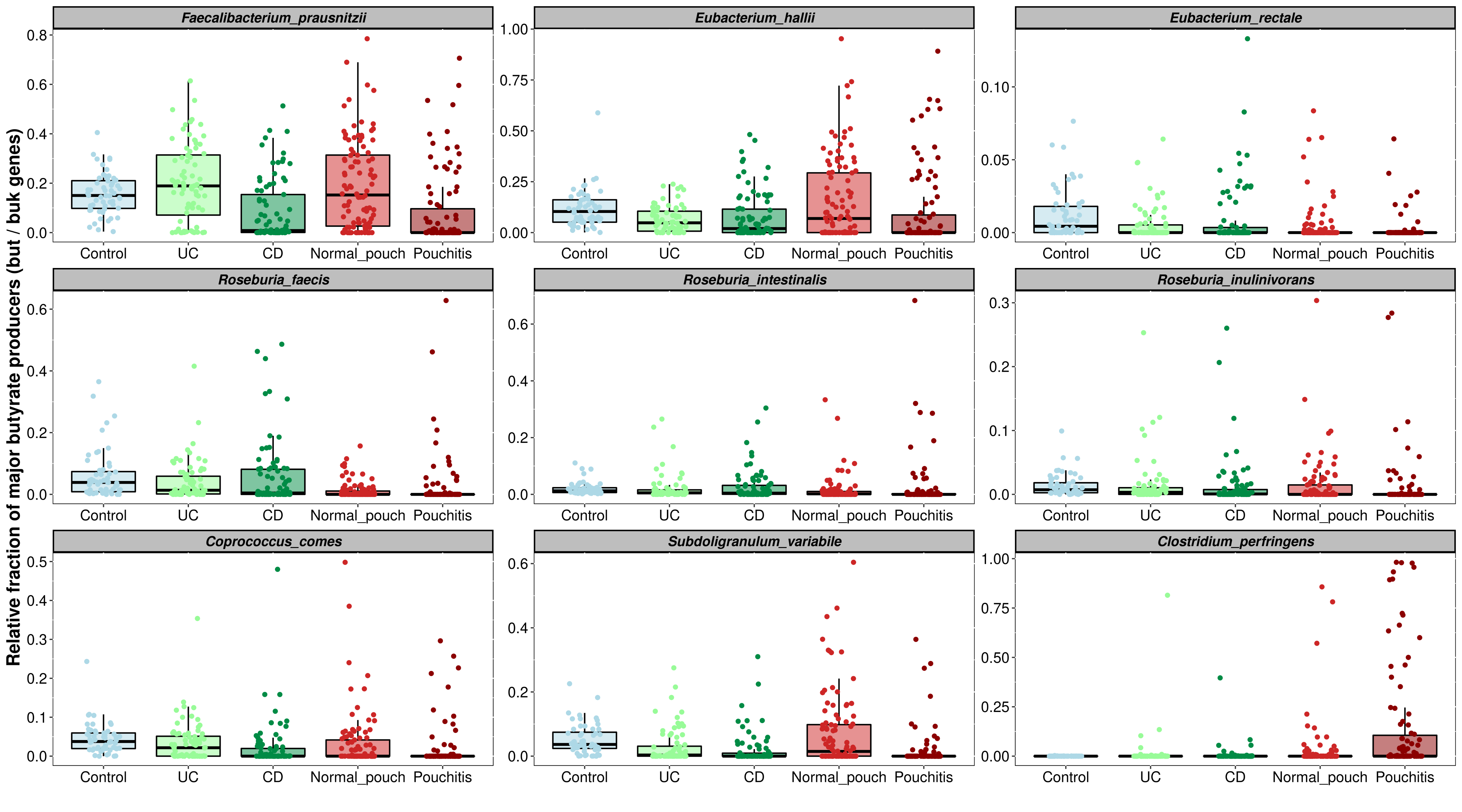

Supplement: FIG S5 [file msystems.00984-20-sf005.tif]

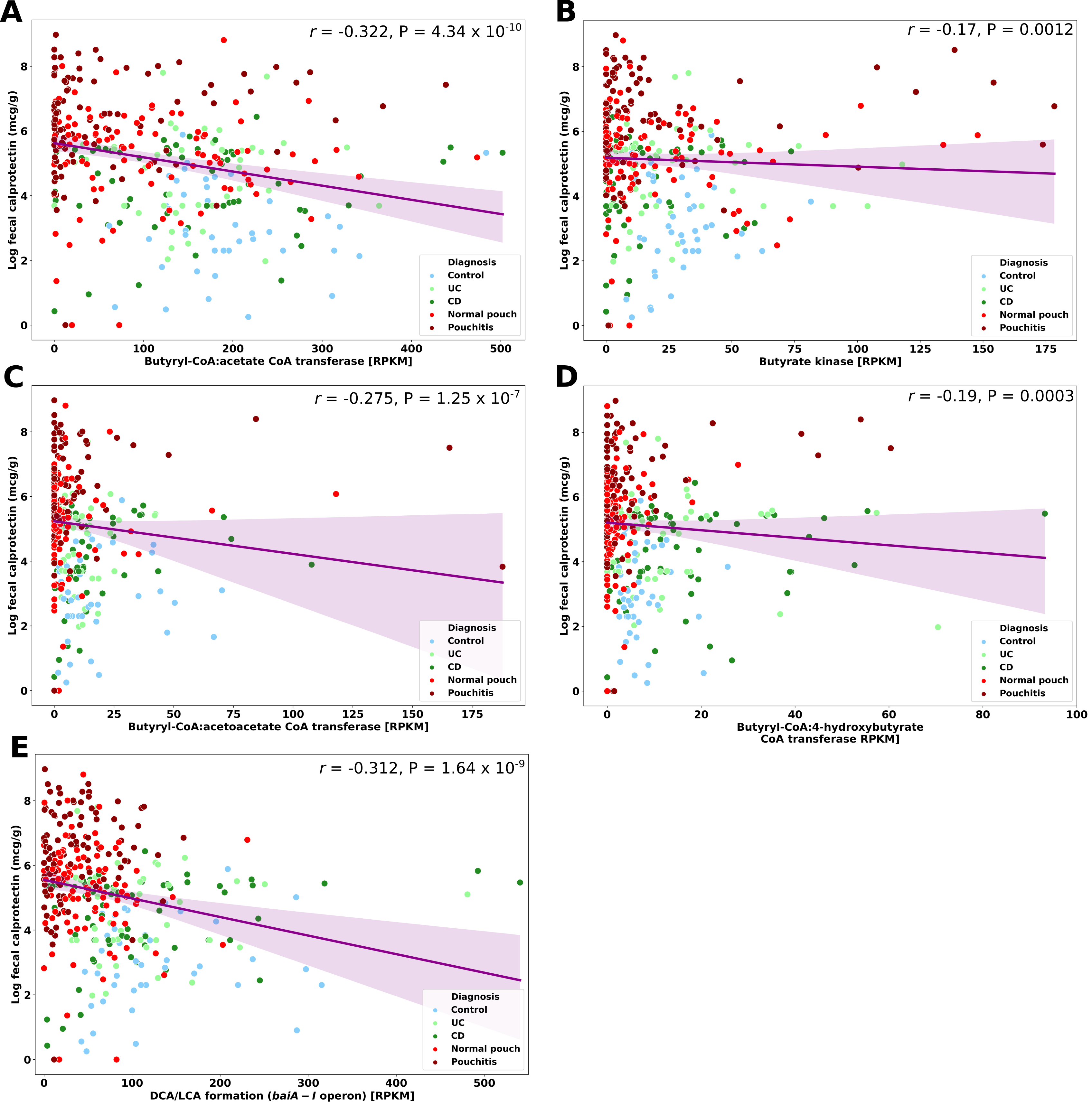

Supplement: FIG S6 [file msystems.00984-20-sf006.tif]

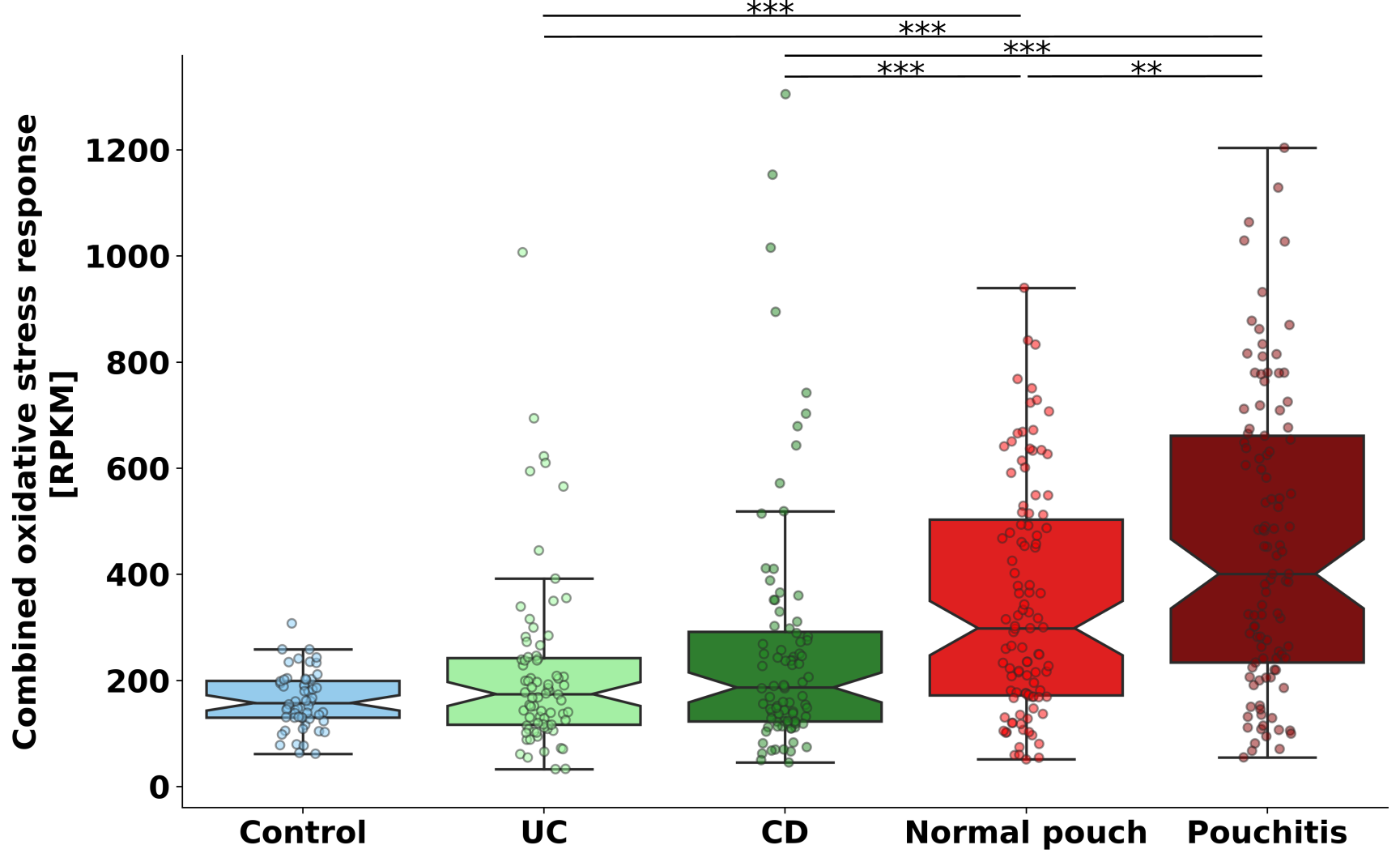

Supplement: FIG S7 [file msystems.00984-20-sf007.tif]

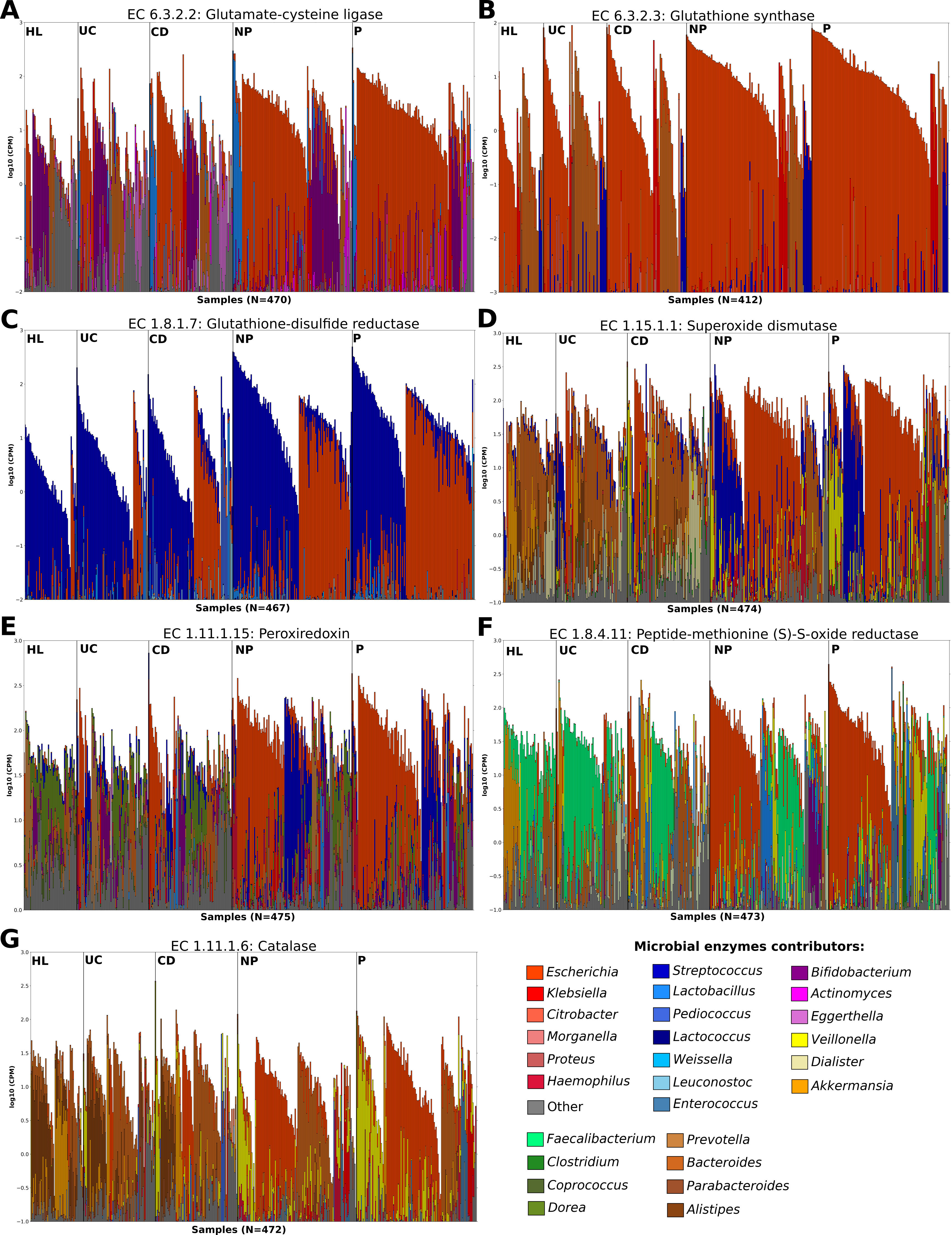

Supplement: FIG S8 [file msystems.00984-20-sf008.jpg]
